# mmVelo: a deep generative model for estimating cell state-dependent dynamics across multiple modalities

**DOI:** 10.1093/bioinformatics/btag652

**Published:** 2026-08-31

**Authors:** Satoshi Nomura, Yasuhiro Kojima, Kodai Minoura, Shuto Hayashi, Ko Abe, Haruka Hirose, Teppei Shimamura

**Affiliations:** Japanese Red Cross Aichi Medical Center, Nagoya Daiichi Hospital, Nagoya, Japan; Laboratory of Computational Life Science, National Cancer Center Research Institute, Tokyo, Japan; Japanese Red Cross Aichi Medical Center, Nagoya Daiichi Hospital, Nagoya, Japan; Department of Computational and Systems Biology, Division of Biological Data Science, Medical Research Laboratory, Institute for Integrated Research, Institute of Science Tokyo, Tokyo, Japan; Department of Computational and Systems Biology, Division of Biological Data Science, Medical Research Laboratory, Institute for Integrated Research, Institute of Science Tokyo, Tokyo, Japan; Department of Computational and Systems Biology, Division of Biological Data Science, Medical Research Laboratory, Institute for Integrated Research, Institute of Science Tokyo, Tokyo, Japan; Japanese Red Cross Aichi Medical Center, Nagoya Daiichi Hospital, Nagoya, Japan; Department of Computational and Systems Biology, Division of Biological Data Science, Medical Research Laboratory, Institute for Integrated Research, Institute of Science Tokyo, Tokyo, Japan

## Abstract

**Motivation:**

Single-cell multiomics reveals regulatory relationships across biological layers but captures only static snapshots, obscuring the dynamics coordinated across modalities. RNA velocity predicts transcriptome dynamics, yet cannot be extended to other layers such as the regulome, leaving chromatin accessibility dynamics unresolved.

**Results:**

We developed mmVelo (multimodal velocity of single cells), a deep generative model that infers cell state dynamics from spliced and unspliced mRNA and projects them onto other modalities, yielding chromatin velocity at single-peak resolution. In developing mouse brain, mmVelo accurately recovered accessibility dynamics; in mouse skin, it identified transcription factors regulating accessibility. Decomposing posterior velocity variability into manifold-aligned and off-manifold components revealed modality-specific uncertainty structure, with chromatin fluctuation elevated near lineage branching. Using multiomics data as a bridge, mmVelo inferred the dynamics of missing modalities from single-modal human brain data.

**Availability and implementation:**

Source code is freely available under the MIT license at https://github.com/nomuhyooon/mmVelo; the version and test data used here are archived at https://doi.org/10.5281/zenodo.20103609.

## 1 Introduction

Single-cell multiomics technologies have emerged as powerful tools for investigating regulatory relationships within individual cells (Baysoy *et al.* 2023, Vandereyken *et al.* 2023). These technologies facilitate simultaneous measurement of multiple modalities from a single cell, providing a comprehensive view of the complex interactions between different molecular layers, including the transcriptome, proteome, and regulome (Angermueller *et al.* 2016, Hou *et al.* 2016, Hu *et al.* 2016, Cao *et al.* 2018, Clark *et al.* 2018, Liu *et al.* 2019, Mimitou *et al.* 2019, Rooijers *et al.* 2019, Xing *et al.* 2020, Mimitou *et al.* 2021, Plongthongkum *et al.* 2021, Sun *et al.* 2021, Zhu *et al.* 2021, Swanson *et al.* 2021, Yan *et al.* 2021, Wang *et al.* 2021, Chen *et al.* 2022, Luo *et al.* 2022, Pan *et al.* 2022, Rang *et al.* 2022, Xu *et al.* 2022). Specifically, methods such as SNARE-seq (Chen *et al.* 2019), Paired-seq (Zhu *et al.* 2019), SHARE-seq (Ma *et al.* 2020c), and 10× Genomics multiome enable the concurrent measurement of gene expression (scRNA-seq; single-cell RNA sequencing) and chromatin accessibility (scATAC-seq; single-cell assay for transposase accessible chromatin with high-throughput sequencing), thereby advancing our understanding of transcriptional regulatory mechanisms. Importantly, regulatory relationships are inherently dynamic and require the incorporation of temporal information from each modality for accurate estimation. Regulatory networks within cells have been estimated using time-series or ordinary differentiation equations (Gardner *et al.* 2003, Di Bernardo et al. 2004, Bansal *et al.* 2006, Wu *et al.* 2014, Ocone *et al.* 2015, Matsumoto *et al.* 2017, Ma *et al.* 2020a). However, owing to the destructive nature of sequencing experiments, single-cell data represent static snapshots at the time of measurement, hindering evaluation of the coordinated dynamic changes across modalities (Weinreb *et al.* 2018).

Computational approaches such as trajectory inference and RNA velocity have been developed to capture temporal dynamics in single-cell measurements. Trajectory inference algorithms construct graphs based on cell similarity and assign a pseudotime to each cell, placing the cells along a one-dimensional timeline that represents the progression of a biological process (Trapnell *et al.* 2014, Cannoodt *et al.* 2016, Haghverdi *et al.* 2016, Setty *et al.* 2016, 2019, Saelens *et al.* 2019). However, for graph construction, trajectory directionality needs to be known and changes in cell states that occur in directions orthogonal to a one-dimensional timeline are not captured. In contrast, RNA velocity estimates the time derivative of the gene expression by linking the abundance of unspliced and spliced mRNA using a mathematical model of splicing kinetics, thereby enabling the prediction of future cell states without prior knowledge and is not confined to a one-dimensional timeline (La Manno *et al.* 2018, Bergen *et al.* 2020). However, predicting dynamics based on RNA velocity models is limited to specific modalities in which a mathematical model can be assumed, and corresponding observations such as unspliced and spliced mRNA can be measured (Bergen *et al.* 2021). Extending these temporal predictions to arbitrary modalities other than RNA, such as chromatin accessibility, remains a significant challenge.

Several studies have considered dynamic changes in chromatin accessibility: by incorporating epigenomic data into a differential equation model of gene expression changes over time, MultiVelo extends the RNA velocity framework (Li *et al.* 2023). By adding a differential equation model for changes in chromatin accessibility, the model estimates the dynamics of chromatin accessibility for each gene. However, this aggregates the peak accessibility for each gene, making it difficult to determine the dynamics at the resolution of the single-peak level. Chromatin velocity uses data from simultaneous measurements of heterochromatin and euchromatin, treating them as the relationship between unspliced and spliced mRNA in the RNA velocity framework, to estimate the dynamics of chromatin accessibility (Tedesco *et al.* 2022). This method can be used to estimate changes in chromatin accessibility at the single-peak level. However, it requires specialized measurement techniques, which limits its general applicability. Additionally, gene expression is not measured simultaneously, limiting our understanding of the regulatory mechanisms across multiple molecular layers. Thus, a method that can simultaneously estimate dynamic changes in gene expression and chromatin accessibility at a single-peak resolution has not yet been developed. This poses a significant challenge for elucidating the detailed mechanisms of gene expression regulation.

To address these challenges, we propose mmVelo, a computational framework designed to estimate cell state-dependent dynamics across multiple modalities using single-cell multiomics data. By utilizing splicing kinetics and multimodal representation learning, mmVelo infers cell state dynamics on joint representations and estimates temporal changes in specific modalities by mapping these dynamics, thereby estimating the temporal changes in chromatin accessibility (chromatin velocity). Focusing on gene expression and chromatin accessibility, we demonstrated that mmVelo accurately estimates chromatin velocity at the single-peak resolution. Additionally, by utilizing the estimated chromatin velocity, we showed that it is possible to predict temporal changes in motif enrichment and identify candidate transcription factors (TFs) that regulate chromatin accessibility. Finally, using multiomics data as a bridge, we illustrated that mmVelo enables integrated analysis of single-modality data and allows for the estimation of dynamics in missing modalities, such as predicting chromatin velocity from gene expression data. mmVelo reveals the dynamic interactions between modalities, providing insights into regulatory relationships.

## 2 Methods

### 2.1 Overview of the mmVelo algorithm

mmVelo estimates chromatin accessibility dynamics in three stages. Initially, cell states are inferred by leveraging information from all modalities of multiomics data. Subsequently, cell state dynamics are estimated using cell-wise transcriptional profiles and splicing kinetics. Finally, chromatin accessibility dynamics are estimated by mapping cell state dynamics onto the space of chromatin accessibility. Detailed explanations of each of these processes are provided in the following sections.

### 2.2 Cell state inference using mixture-of-experts multimodal variational autoencoder

First, we infer multimodal cell states using a variational autoencoder (VAE; Kingma and Welling 2013, Lopez *et al.* 2018, Minoura *et al.* 2021). Let *n* index cells, *p* index chromatin accessibility regions, and *g* index genes. We assumed a generative model in which the observed chromatin accessibility, $a_{np}$, along with the gene expression levels of spliced mRNA, $s_{ng}$, and unspliced mRNA, $u_{ng}$, were generated based on the following formula:


$$\begin{array}{cc} z_{n} & ∼\text{Normal}(0,I)\\ l_{n}^{a} & ∼\text{LogNormal}(\mu_{l^{a}},\sigma_{l^{a}}^{2})\\ w_{np}^{a}|z_{n} & ∼\text{Gamma}(f_{p}^{a}(z_{n}),\theta_{p}^{a})\\ y_{np}^{a}|l_{n}^{a},w_{np}^{a} & ∼\text{Poisson}\left(\frac{l_{n}^{a}}{\;\text{exp}(\mu_{l^{a}}+\sigma_{l^{a}}^{2}/2)}C_{p}^{a}w_{np}^{a}\right)\\ h_{np}^{a}|z_{n} & ∼\text{Bernoulli}(g_{p}^{a}(z_{n}))\\ a_{np} & =\left\{ \begin{array}{cc} y_{np}^{a} & \text{if}\;h_{np}^{a}=0\\ 0 & \text{otherwise} \end{array}\right.\\ l_{n}^{r} & ∼\text{LogNormal}(\mu_{l^{r}},\sigma_{l^{r}}^{2})\\ w_{ng}^{s}|z_{n} & ∼\text{Gamma}(f_{g}^{s}(z_{n}),\theta_{g}^{s})\\ s_{ng}|l_{n}^{r},w_{ng}^{s} & ∼\text{Poisson}\left(\frac{l_{n}^{r}}{\;\text{exp}(\mu_{l^{r}}+\sigma_{l^{r}}^{2}/2)}C_{g}^{s}w_{ng}^{s}\right)\\ w_{ng}^{u}|z_{n} & ∼\text{Gamma}(f_{g}^{u}(z_{n}),\theta_{g}^{u})\\ u_{ng}|l_{n}^{r},w_{ng}^{u} & ∼\text{Poisson}\left(\frac{l_{n}^{r}}{\;\text{exp}(\mu_{l^{r}}+\sigma_{l^{r}}^{2}/2)}C_{g}^{u}w_{ng}^{u}\right) \end{array}$$

where $z_{n}$ represents the cell state, assumed to follow a normal distribution. The random variables $l_{n}^{a}$ and $l_{n}^{r}$ correspond to the scaling factors in scATAC-seq and scRNA-seq, respectively, and were assumed to follow a log-normal distribution. The parameters $\mu_{l^{a}}$ and $\sigma_{l^{a}}^{2}$, which denote the mean and variance of the log-normal distribution, respectively, were set to the empirical mean and variance of the log-library size (similarly for $\mu_{l^{r}}$ and $\sigma_{l^{r}}^{2}$ in scRNA-seq). These parameters were tailored to reflect the variability introduced by the differing library sizes among cells. Furthermore, $\theta_{a}^{p}$, $\theta_{g}^{s}$, and $\theta_{g}^{u}$ represent the inverse dispersion parameters for each modality, set for each genomic region and gene and optimized during the training process. $C_{p}^{a}$, $C_{g}^{s}$, and $C_{g}^{u}$ represent the average accessibility scale and average expression scale for peak *p* and gene *g*, respectively. These values were calculated based on the observed counts as $C_{p}^{a}=E[a_{p}|a_{p}>0]$ for chromatin accessibility and analogously for spliced and unspliced mRNA. The functions $f_{p}^{a}$, $f_{g}^{s}$, and $f_{g}^{u}$ are the decoder neural networks corresponding to each modality. These networks take the cell state $z_{n}$ as the input, and output the mean chromatin accessibility or gene expression for the respective cell. $g_{p}^{a}$ outputs the probability of dropout for a given element, which is a functionality employed to account for the sparsity observed in chromatin accessibility measurements. This formulation is particularly important in our setting because chromatin dynamics are modeled at single-peak resolution rather than after gene-level aggregation, making sparsity and noise more pronounced.

Given the difficulty in analytically deriving the posterior distributions of the latent variables $z_{n}$, $l_{n}^{a}$, and $l_{n}^{r}$, we assume an approximate posterior distribution parameterized by neural networks that take observed values as inputs. This approximation is optimized through variational inference, as follows:


$$q(z_{n},l_{n}^{a},l_{n}^{r}|a_{n},s_{n},u_{n})=q(z_{n}|a_{n},s_{n},u_{n})q(l_{n}^{a}|a_{n})q(l_{n}^{r}|s_{n},u_{n})$$

Here, the variational posterior distribution of the cell state is represented as a mixture of experts from the variational posterior distributions of each modality (Shi *et al.* 2019, Minoura *et al.* 2021), as


$$q(z_{n}|a_{n},s_{n},u_{n})=\frac{1}{2}(q(z_{n}|a_{n})+q(z_{n}|s_{n},u_{n}))$$

For the variational distribution of the cell state and scaling factor in each modality, we assumed a normal and a log-normal distribution, respectively. The means and variances of these distributions are provided by encoder neural networks that use each modality as an input. Since $p(a_{n}|z_{n},l_{n}^{a})$, $p(s_{n}|z_{n},l_{n}^{r})$, and $p(u_{n}|z_{n},l_{n}^{r})$ have closed-form probability density functions, the latent variables $w_{np}^{a}$, $y_{np}^{a}$, and $h_{np}^{a}$ for scATAC-seq, $w_{ng}^{s}$ and $y_{ng}^{s}$ for spliced mRNA, as well as $w_{ng}^{u}$ and $y_{ng}^{u}$ for unspliced mRNA, can be integrated out (Lopez *et al.* 2018). Specifically, $p(a_{n}|z_{n},l_{n}^{a})$ follows a zero-inflated negative binomial distribution with a mean of $\frac{l_{n}^{a}}{\;\text{exp}(\mu_{l^{a}}+\sigma_{l^{a}}^{2}/2)}C_{p}^{a}f_{p}^{a}(z_{n})$, a peak-specific dispersion $\theta_{p}^{a}$, and a zero-inflation probability $g_{p}^{a}(z_{n})$, while $p(s_{n}|z_{n},l_{n}^{r})$ and $p(u_{n}|z_{n},l_{n}^{r})$ follow negative binomial distributions with means $\frac{l_{n}^{r}}{\;\text{exp}(\mu_{l^{r}}+\sigma_{l^{r}}^{2}/2)}C_{g}^{s}f_{g}^{s}(z_{n})$ and $\frac{l_{n}^{r}}{\;\text{exp}(\mu_{l^{r}}+\sigma_{l^{r}}^{2}/2)}C_{g}^{u}f_{g}^{u}(z_{n})$, and gene-specific dispersions $\theta_{g}^{s}$ and $\theta_{g}^{u}$, respectively. The model parameters were optimized by maximizing the variational lower bound of the log likelihood:


(1) $$\begin{array}{c} \begin{array}{c} \text{log}\;p(a_{n},s_{n},u_{n})\geq E_{q(z_{n},l_{n}^{a},l_{n}^{r}|a_{n},s_{n},u_{n})}\left[\text{log}\;\frac{p(z_{n},l_{n}^{a},l_{n}^{r}a_{n},s_{n},u_{n})}{q(z_{n},l_{n}^{a},l_{n}^{r}|a_{n},s_{n},u_{n})}\right] \end{array}\\ \begin{array}{cc} & =E_{q(z_{n},l_{n}^{a},l_{n}^{r}|a_{n},s_{n},u_{n})}[\text{log}\;p(a_{n}|z_{n},l_{n}^{a})+\text{log}\;p(s_{n},u_{n}|z_{n},l_{n}^{r})]\\ & -\text{KL}[q(z_{n}|a_{n},s_{n},u_{n})||p(z_{n})]\\ & -\text{KL}[q(l_{n}^{a}|a_{n})||p(l_{n}^{a})]-\text{KL}[q(l_{n}^{r}|s_{n},u_{n})||p(l_{n}^{r})] \end{array} \end{array}$$

where stratified sampling (Robert *et al.* 1999) is employed for the first term on the right-hand side of equation (1) during training.

### 2.3 Fine-tuning decoders for smoothed profile reconstruction

After estimating the cell states through the aforementioned process, mmVelo was fine-tuned to predict the smoothed profiles $\mu_{np}^{a}$, $\mu_{ng}^{s}$, and $\mu_{ng}^{u}$ for chromatin accessibility and spliced/unspliced mRNA. The smoothed profiles were calculated based on the neighborhood relationships of the cell states. Specifically, they were determined by taking the average of the size-corrected (to 10 000, Ahlmann-Eltze and Huber 2023) log1p-normalized counts among the *k*-nearest neighbor cells. Here, we set k=50. Furthermore, a Gaussian distribution was assumed for the smoothed profiles:


$$\begin{array}{c} \mu_{np}^{a}|z_{n}∼\text{Normal}(C_{p}^{a}f_{p}^{a}(z_{n}),\sigma_{ap}^{2})\\ \mu_{ng}^{s}|z_{n}∼\text{Normal}(C_{g}^{s}f_{g}^{s}(z_{n}),\sigma_{sg}^{2})\\ \mu_{ng}^{u}|z_{n}∼\text{Normal}(C_{g}^{u}f_{g}^{u}(z_{n}),\sigma_{ug}^{2}) \end{array}$$

where $\sigma_{ap}$, $\sigma_{sg}$, and $\sigma_{ug}$ are the standard deviations set for each genomic region and gene and are optimized during training process. The model parameters were optimized by maximizing the following term (applied only to the decoder, with all the other parameters of the model held fixed):


$$E_{q(z_{n}|a_{n},s_{n},u_{n})}[\text{log}\;p(\mu_{n}^{a},\mu_{n}^{s},\mu_{n}^{u}|z_{n})]$$

### 2.4 Inference of cell state dynamics

Next, mmVelo inferred cell state dynamics consistent with changes in gene expression described by the RNA velocity equation. This concept was inspired by Nagaharu *et al.* (2022), on which our original formulations were based. At this stage, we assume that the temporal change in the spliced mRNA $ds_{n}/dt$ can be obtained from the following generative model:


$$\begin{array}{c} z_{n}∼\text{Normal}(0,I)\\ d_{n}∼\text{Normal}(0,I)\\ \frac{ds_{n}}{dt}|z_{n},d_{n}∼\text{vMF}\left(\frac{\text{tanh}(C^{s}⊙(f^{s}(z_{n}+\rho d_{n})-f^{s}(z_{n})))}{||\text{tanh}(C^{s}⊙(f^{s}(z_{n}+\rho d_{n})-f^{s}(z_{n})))|{|}_{2}},\kappa \right) \end{array}$$

where ⊙ denotes the Hadamard product, $\rho d_{n}$ (ρ≪1) represents the transition of the cell state over a short time interval, and $d_{n}$ is assumed to follow a normal distribution. In this study, we set ρ=0.01 to ensure that $\rho d_{n}$ represents a sufficiently small transition in the cell state space. $ds_{n}/dt$ represents the temporal change in the spliced mRNA and is assumed to follow the von Mises–Fisher distribution. The mean of $ds_{n}/dt$ was determined by the difference between the expression profiles before and after a short time interval. The difference in expression profiles was obtained by assuming that the current cellular state is represented by $z_{n}$ and, after a short time interval, by $z_{n}+\rho d_{n}$, and mapping these states into the gene expression space through the decoder $f^{s}$ which outputs the average expression profile of spliced mRNA. κ is the concentration parameter of the distribution. In this study, we assumed κ=1.

Owing to the difficulty in analytically deriving the posterior distributions of the latent variables $z_{n}$ and $d_{n}$, we inferred these through variational inference. During this process, we assume that the variational distribution of the cell state transition $d_{n}$ is dependent on the cell state $z_{n}$, and is determined as follows:


$$q(z_{n},d_{n}|a_{n},s_{n},u_{n})=q(z_{n}|a_{n},s_{n},u_{n})q(d_{n}|z_{n})$$

Here, we assumed a normal distribution for the variational posterior distribution of $d_{n}$, with the mean and variance of this distribution parameterized by an encoder that utilizes the cell state $z_{n}$ as input data. Given the observed values of $ds_{n}/dt$, optimization was achieved by maximizing the variational lower bound of the log likelihood:


$$\begin{array}{cc} \text{log}\;p\left(\frac{ds_{n}}{dt}\right) & \geq E_{q(z_{n},d_{n}|a_{n},s_{n},u_{n})}\left[\text{log}\;\frac{p\left(z_{n},d_{n},\frac{ds_{n}}{dt}\right)}{q(z_{n},d_{n},|a_{n},s_{n},u_{n})}\right]\\ & =E_{q(z_{n}|a_{n},s_{n},u_{n})q(d_{n}|z_{n})}\left[\text{log}\;p\left(\frac{ds_{n}}{dt}|z_{n},d_{n}\right)\right]\\ & -\text{KL}[q(z_{n}|a_{n},s_{n},u_{n})||p(z_{n})]\\ & -E_{q(z_{n}|a_{n},s_{n},u_{n})}[\text{KL}[q(d_{n}|z_{n})||p(d_{n})]] \end{array}$$

During optimization, we assumed that $ds_{n}/dt_{\mathrm{obs}}$, the observed values of $ds_{n}/dt$ were provided based on the assumptions of the splicing kinetics model (La Manno *et al.* 2018) and the output values of the decoders for spliced and unspliced mRNA, $f_{w}^{s}(z_{n})$ and $f_{w}^{u}(z_{n})$, as given by the following equation:


$$\frac{ds_{n}}{dt_{\mathrm{obs}}}=\frac{\text{tanh}(\beta ⊙C^{u}⊙f^{u}(z_{n})-\gamma ⊙C^{s}⊙f^{s}(z_{n}))}{||\text{tanh}(\beta ⊙C^{u}⊙f^{u}(z_{n})-\gamma ⊙C^{s}⊙f^{s}(z_{n}))|{|}_{2}}$$

where $\beta \in R^{g}$ is a vector containing the splicing rates of each gene, and $\gamma \in R^{g}$ is a vector containing the degradation rates of each gene. These were set as learnable parameters and optimized during the training process. The symbol ⊙ denotes the Hadamard product. To prevent genes with large changes in expression levels from being disproportionately emphasized in the inference procedure, the scaling of change in each gene’s expression was performed using the tanh function. During the optimization of the cell state dynamics, all model parameters except the encoder for $d_{n}$ and the splicing kinetics parameters (β,γ) were fixed.

### 2.5 Estimation of chromatin velocity

Using the model learned through the above process, we estimated chromatin velocity, the temporal change in chromatin accessibility (ΔATAC). This estimation is achieved by mapping the cellular states during a micro duration to the chromatin accessibility space and calculating their differences, as follows:


$$\Delta \text{ATAC}=C^{a}⊙(f^{a}(z_{n}+\rho d_{n})-f^{a}(z_{n}))$$

In addition, we estimated the temporal changes in unspliced mRNA (Δunspliced) and spliced mRNA (Δspliced) as follows:


$$\begin{array}{c} \Delta \text{unspliced}=C^{u}⊙(f^{u}(z_{n}+\rho d_{n})-f^{u}(z_{n}))\\ \Delta \text{spliced}=C^{s}⊙(f^{s}(z_{n}+\rho d_{n})-f^{s}(z_{n})) \end{array}$$

### 2.6 Training procedure

We trained mmVelo on 90% of the data and used 10% as the validation set. By default, mmVelo was optimized using AdamW (Loshchilov and Hutter 2017) with a learning rate of 0.0001 for the inference of the cell state and cell state dynamics, and 0.01 for decoder fine-tuning to reconstruct the smoothed profiles. We used a minibatch size of 128 and trained the model for 500 epochs. For each procedure, training was stopped early if there was no improvement in the ELBO (evidence lower bound) on the validation dataset for 30 epochs. As done in a previous study (Minoura *et al.* 2021), for cell state inference, we down-weighted the KL (Kullback-Leibler) divergence between the cell state variational posterior and its prior for the first 30 epochs.

To ensure that the genes used for estimation had comparable information content in both spliced and unspliced mRNA, we excluded genes with an observed total count ratio of spliced to unspliced mRNA less than 1/50 or greater than 50 during the optimization of cell state dynamics. Additionally, the initial values of the splicing mathematical model parameters β and γ were calculated based on the steady-state model of RNA velocity (La Manno *et al.* 2018). Moreover, the squared error of the deviation from the initial values of β and γ was included as a regularization term in the optimization objective.

Hyperparameters, including the number of hidden layers, hidden layer dimensionality, latent dimensionality, and learning rate, were selected using Optuna (Akiba *et al.* 2019), with the ELBO on the validation set as the objective function. The selected hyperparameter values used in all experiments are reported in the code repository (https://github.com/nomuhyooon/mmVelo, https://doi.org/10.5281/zenodo.20103609). For initialization, prior mean parameters for the latent variables were set to zero. The learnable scale parameter for the transition prior was initialized so that its softplus-transformed value was 1 at the start of training. Dispersion- and variance-related parameters were initialized from a normal distribution. Other parameters were initialized using the default implementation settings and optimized during training.

### 2.7 Sensitivity analysis of latent dimensionality and smoothing

To assess the sensitivity of mmVelo to the latent dimensionality of the cell-state representation and to the use of smoothing, we performed an ablation analysis on the embryonic mouse brain and SHARE-seq mouse skin hair follicle datasets. We evaluated latent dimensions of 5, 10, 30, and 50, and additionally considered a no-smoothing condition at latent dimension 10. All other model settings were kept unchanged unless otherwise noted.

For each setting, we estimated chromatin velocity and evaluated the inferred vector field using the vector consistency score (VCS) defined by the following equation:


$$\sum_{t}\sum_{c\in C_{t}}\left({\bar{X}}_{t+1}-{\bar{X}}_{t}\right)\frac{\;\text{sgn}(v_{c})}{\left|C_{t}\right|},\;{\bar{X}}_{t}=\frac{1}{\left|C_{t}\right|}\sum_{c\in C_{t}}x_{c}$$

Here, $x_{c}$ represents the log-normalized observed count in cell *c*, normalized by dividing the observed count by $\text{max}(x_{c})$ to scale the expression. $v_{c}$ denotes the estimated velocity in cell *c*, *t* refers to pseudotime bins, and $C_{t}$ represents the set of cells within pseudotime bin *t*.

We additionally visualized the inferred chromatin velocity fields using streamline plots to assess whether the global flow pattern was consistent with the expected biological directionality. Based on these quantitative and qualitative comparisons, we selected latent dimension 10 with smoothing as the default configuration used in the main analyses.

We note that, in the SHARE-seq hair follicle dataset, the VCS was computed using a single pseudotime axis despite the presence of multiple lineages. Therefore, in this dataset, the VCS should be interpreted as a complementary summary statistic rather than as a complete measure of performance, and was considered together with the streamline-based assessment of biological plausibility.

### 2.8 Benchmarking against velocity estimation methods

To benchmark the accuracy of the estimated velocity, we conducted a quantitative comparison of velocity consistency. To this end, we used hair shaft-cuticle/cortex lineage cells during mouse hair follicle development. These cells were chosen because (i) they offer a sufficient number of cells for quantitative evaluation and (ii) their expansion in directions orthogonal to differentiation is relatively modest. Pseudotime for the hair shaft-cuticle/cortex lineage cells was inferred using diffusion pseudotime (Haghverdi *et al.* 2016), and the cell population was subsequently divided into 10 pseudotime bins. Based on these bins, the accuracy of the velocity estimates for each gene or peak was quantified for each method using the velocity consistency score described in the previous section.

For benchmarking, we selected scVelo and MultiVelo (Bergen *et al.* 2020, Li *et al.* 2023). To ensure a fair comparison, the neighborhood relationships among cells were computed based on cell states similar to mmVelo, which were estimated using the VAE module of mmVelo. These relationships were then used to smooth the profiles provided as inputs to scVelo and MultiVelo.

### 2.9 Calculation of motif velocity

To estimate the motif velocity, we expanded upon the motif activity concept, as proposed in chromVAR (Schep *et al.* 2017). Let $y_{n}$ be the motif activity of a motif in cell *n*, and $dy_{n}/dt$ be the corresponding motif velocity. Initially, the motif activity was calculated in a manner similar to chromVAR based on the following equation:


$$y_{n}=\frac{a{\left(z_{n}\right)}^{T}m-e_{n}^{T}m}{e_{n}^{T}m}$$

where $a(z_{n})$ represents the reconstructed chromatin accessibility profiles of cell *n*, $e_{n}$ is the expected value of the read count fragments in cell *n*, and m is a binary vector indicating the presence of a motif at individual peaks, derermined using the motifmatchr package (Schep *et al.* 2017). Specifically, $e_{n}$ is the row vector of E, where $E_{ij}$ is calculated as $E_{ij}=\sum_{p}a_{ip}(z_{i})\sum_{n}a_{nj}(z_{n})/\sum_{n}\sum_{p}a_{np}(z_{n})$. Although chromVAR accounts for technical biases using background peaks, we did not include this correction for this term because we used smoothed profiles and did not observe these biases. Based on this, the motif velocity was calculated using the following formula:


$$\frac{dy_{n}}{dt}={\left(\frac{dy_{n}}{dz_{n}}\right)}^{T}\frac{dz_{n}}{dt}={\left(\frac{dy_{n}}{dz_{n}}\right)}^{T}d_{n}$$

The position-frequency matrix for each TF motif was downloaded from JASPAR2022 (Castro-Mondragon *et al.* 2022), using the getMatrixSet function in Signac (Stuart *et al.* 2021). TF motifs with corresponding TF mRNA expression were used for analysis.

### 2.10 Quantification of velocity uncertainty

To quantify uncertainty in the velocity estimates produced by mmVelo, we focused on posterior variability in the latent dynamics variable while fixing the latent cell state at its posterior mean. For each cell *n*, we first computed the posterior mean latent cell state


$$\hat{z_{n}}=E[q(z_{n}|a_{n},s_{n},u_{n})].$$

We then drew *S* posterior samples of latent dynamics conditional on the inferred cell state:


$$d_{n}^{\left(s\right)}∼q(d_{n}|\hat{z_{n}}),\;s=1,\ldots ,S.$$

Using these samples, we computed modality-specific posterior velocity samples:


$$v_{n}{\;}^{\text{RNA},(s)}=C_{s}⊙\left(f^{s}(\hat{z_{n}}+\rho d_{n}{\;}^{\left(s\right)})-f^{s}(\hat{z_{n}})\right),$$

$$v_{n}{\;}^{\text{ATAC},(s)}=C_{a}⊙\left(f^{a}(\hat{z_{n}}+\rho d_{n}{\;}^{\left(s\right)})-f^{a}(\hat{z_{n}})\right).$$

For each modality m∈RNA,ATAC, we defined the posterior mean velocity


$$\bar{v_{n}^{m}}=\frac{1}{S}\sum_{s=1}^{S}v_{n}{\;}^{m,(s)}.$$

We quantified the total velocity uncertainty in each modality as the variance of the cosine similarity between posterior velocity samples and the posterior mean velocity:


$$U_{n,\text{total}}^{m}={\text{Var}}_{s}\left[\text{cos}\;\left(v_{n}{\;}^{m,(s)},{\bar{v_{n}}}^{m}\right)\right].$$

To distinguish biologically plausible variability from instability, we further decomposed modality-specific velocity samples into on-manifold and off-manifold components in each modality space.

For each modality *m*, we constructed a local tangent space around cell *n* using the neighborhood of its latent representation. Specifically, letting


$$x_{n}{\;}^{\text{RNA}}=f^{s}(\hat{z_{n}}),\;x_{n}{\;}^{\text{ATAC}}=f^{a}(\hat{z_{n}}),$$

we formed centered neighborhood vectors with the *k*-nearest neighbors $N_{m}(n)$ of cell *n*


$$x_{j}^{m}-x_{n}^{m}|j\in N_{m}(n).$$

We then performed principal component analysis for RNA and truncated singular value decomposition (latent semantic indexing [LSI]) for assay for transposase-accessible chromatin (ATAC) on these local neighborhood vectors and used the top $r_{m}$ principal directions (principal components for RNA and leading singular vectors for ATAC) to define the local tangent basis


$$U_{n}^{m}=[u_{n1}^{m},\ldots ,u_{nr_{m}}^{m}].$$

The corresponding projection matrix is


$$P_{n}^{m}=U_{n}^{m}{\left(U_{n}^{m}\right)}^{⊤}.$$

Each posterior velocity sample was then decomposed into an on-manifold component and an off-manifold component:


$$v_{n,∥}^{m,(s)}=P_{n}^{m}v_{n}^{m,(s)},\;v_{n,⊥}^{m,(s)}=(I-P_{n}^{m})v_{n}^{m,(s)}.$$

For the on-manifold component, we defined the mean projected velocity


$${\bar{v}}_{n,∥}^{m}=\frac{1}{S}\sum_{s=1}^{S}v_{n,∥}^{m,(s)}$$

and quantified on-manifold fluctuation as


$$U_{n,∥}^{m}={\text{Var}}_{s}\left[\text{cos}\;\left(v_{n,∥}^{m,(s)},{\bar{v}}_{n,∥}^{m}\right)\right].$$

For the off-manifold component, we defined the mean orthogonal velocity


$${\bar{v}}_{n,⊥}^{m}=\frac{1}{S}\sum_{s=1}^{S}v_{n,⊥}^{m,(s)}$$

and quantified off-manifold instability as


$$U_{n,⊥}^{m}={\text{Var}}_{s}\left[\text{cos}\;\left(v_{n,⊥}^{m,(s)},{\bar{v}}_{n,⊥}^{m}\right)\right].$$

In this framework, $U_{n,\text{total}}^{m}$ measures the overall uncertainty of modality-specific velocity induced by posterior variability in latent dynamics, $U_{n,∥}^{m}$ captures variability aligned with the local manifold and is interpreted as on-manifold fluctuation, and $U_{n,⊥}^{m}$ captures variability orthogonal to the local manifold and is interpreted as off-manifold instability.

### 2.11 Inferring TF-peak regulatory relationships using chromatin velocity

To identify the TFs that regulate chromatin accessibility, we integrated the SCENIC + framework (Bravo González-Blas *et al.* 2023) with the estimated chromatin velocity using mmVelo to infer regulatory relationships. Initially, to identify covarying peak clusters, we conducted Leiden clustering of peaks based on chromatin velocity with a resolution of 0.8, employing cosine similarity as a metric for the similarity between peaks. Subsequently, using pycisTarget within the SCENIC + framework, we conducted a motif search for each peak cluster and designated the corresponding TFs as putative regulators. Finally, we applied GRNBoost2 (Moerman *et al.* 2019) to regress chromatin velocity from the spliced mRNA expression of putative regulator TFs and calculated the feature importance scores for each TF.

To statistically determine the presence of regulatory relationships based on the feature importance scores, we conducted a permutation test. To obtain the empirical distribution of background importance scores, we permuted the TF expression matrix in the direction of cells (while we did not shuffle in the direction of genes to preserve the correlation structure between genes) and applied GRNBoost2; this procedure was repeated five times. Subsequently, adjustments were made using the Benjamini-Hochberg method (Benjamini and Hochberg 1995), and regulatory relationships were extracted at an FDR of 0.001.

### 2.12 Velocity inference on missing modality

To estimate velocity in the missing modality from single-modality data (scATAC-seq or scRNA-seq), we expanded the mmVelo framework. We used multiome (snATAC-seq + snRNA-seq), scATAC-seq, and scRNA-seq data as input data. The generative and variational models of this hybrid framework were adopted from the mmVelo model.

We first pretrained mmVelo using paired multiome data to establish a shared latent representation across RNA and chromatin accessibility. This pretraining step allows the model to learn a multimodal cell-state space anchored by cells in which both modalities are jointly observed. We then trained the model jointly using paired multiome cells together with unpaired scRNA-seq and scATAC-seq cells.

During this stage, we employed stratified sampling so that cells from each data source were represented during optimization, and the reconstruction loss for each cell was computed only for the modality that was actually observed. Thus, for paired multiome cells, losses from both RNA and chromatin accessibility were included, whereas for unpaired single-modality cells, only the corresponding modality-specific loss was evaluated. This design allows all cells to contribute to learning the shared latent representation without requiring artificial reconstruction targets for unobserved modalities.

To align latent representations across modalities and datasets, we introduced a maximum mean discrepancy term following the strategy used in MultiVI (Ashuach *et al.* 2023). In addition, a conditional one-hot vector encoding dataset/domain information was provided to the encoders and decoders, and a domain adaptation penalty (Ganin *et al.* 2016) was used to further reduce domain-specific distortion in the shared latent space. Together, these components encourage cells from related biological states to occupy nearby regions of latent space even when the measurements are unpaired.

Once unpaired single-modality cells were mapped into the shared latent space learned from multiome data, missing-modality profiles were inferred through the modality-specific decoders. These inferred profiles were then used to estimate the corresponding missing-modality velocity in the same manner as for paired data.

In this setting, neighborhood smoothing was applied only among cells measured in the corresponding modality. Thus, smoothing was not used to directly align unpaired modalities, but rather to stabilize modality-specific profiles against sparsity and local noise. While this procedure can improve robustness, it may also blur biological and batch-specific variation and therefore should be interpreted with caution.

The missing-modality framework is best suited to datasets that represent the same or closely matched biological process across modalities. In the human cortical development experiment presented here, multiome, scRNA-seq, and scATAC-seq data were all obtained from the same developmental stage [post-conceptional week (PCW21)], providing a reasonable basis for this shared-process assumption. When modality-specific cell populations are not shared across datasets, cross-modal inference becomes an extrapolation problem, and the inferred profiles and velocities should be interpreted cautiously. Likewise, although latent alignment encourages biological integration, it does not fully guarantee separation of batch effects from true biological differences in unpaired settings.

### 2.13 Preprocessing of data

#### 2.13.1 10× embryonic E18 mouse brain

The filtered accessibility matrix for ATAC-seq and position-sorted RNA alignment (BAM) file of E18 mouse embryonic brain data were downloaded from 10× Genomics website. Unspliced and spliced RNA reads were quantified using the Velocyto run 10× command (La Manno *et al.* 2018). Genes with a minimum expression level of fewer than 10 counts were excluded. To focus our analysis on actively differentiating cell populations, we initially excluded interneurons, Cajal–Retzius, and microglia cells. We performed gene and cell filtering, clustering, and annotation based on marker gene expression using the standard Scanpy pipeline (Wolf *et al.* 2018). After filtering out cells that did not actively differentiate, the gene expression count data were normalized using the SCTransform function (Hafemeister and Satija 2019), implemented in Seurat (note that this normalized expression profile was used only for feature preprocessing and not as model inputs). Following normalization, the top 3000 highly variable genes were selected. For ATAC-seq data, peaks were filtered based on their proximity to transcription start sites (TSS) and their correlation with gene expression. This peak filtering strategy was inspired by the concept proposed in the SHARE-seq study (Ma *et al.* 2020c): the LinkPeaks function implemented in Signac (Stuart *et al.* 2021) was applied around TSS regions (±50kb) to identify peaks associated with gene expression. Genes exhibiting a significant correlation (*P* < .05) with at least five peaks were identified as domains of regulatory chromatin (DORC) genes, and these genes were added to the list of RNA-seq genes. Next, the LinkPeaks function was applied within ± 500 kb of the TSS, and peaks showing high absolute correlation values (>0.01) with any of the RNA-seq genes were selected as features. The resulting filtered count matrix of gene expression and chromatin accessibility was used as the input for mmVelo. This filtering procedure reduces the influence of weakly informative or noisy peaks, which is particularly important when chromatin dynamics are modeled at single-peak resolution. In this setting, the effective accessibility support for a gene can be quantified by the number of significantly associated peaks retained after filtering.

#### 2.13.2 SHARE-seq mouse skin (hair follicle) data

The quantified ATAC-seq expression matrix, RNA alignment BAM file, and cell annotations of the SHARE-seq mouse skin dataset were downloaded from the Gene Expression Omnibus (GEO). Unspliced and spliced RNA reads were quantified using the Velocyto run command. Hair follicle cells (TAC, IRS, medulla, and hair shaft-cuticle/cortex) were extracted based on cell annotations. Genes with a minimum expression level of fewer than 10 counts were excluded. Gene expression count data were normalized using the SCTransform function implemented in Seurat (note that this normalized expression profile was used only for feature preprocessing). Following normalization, the top 3000 highly variable genes were selected. For ATAC-seq data, peaks were filtered based on their proximity to TSS and their correlation with gene expression. First, the LinkPeaks function implemented in Signac was applied around TSS regions (±50kb) to identify peaks associated with gene expression. Genes exhibiting a significant correlation (*P* < .05) with at least 10 peaks were identified as DORC genes, and those genes were added to the list of RNA-seq genes. Next, the LinkPeaks function was applied within ± 500 kb of the TSS, and the top 25 000 peaks showing high absolute correlation values (>0.01) with any of the RNA-seq genes were selected as features. The resulting filtered count matrix of gene expression and chromatin accessibility was used as the input for mmVelo.

#### 2.13.3 Human cerebral cortex

The quantified ATAC-seq expression matrix, quantified unspliced and spliced count matrix, and cell annotations were downloaded from the GEO. We initially excluded interneurons, pericytes, endothelial cells, and microglia cells, because these cells do not actively differentiate. Genes with a minimum expression level of fewer than 10 counts were excluded. Gene expression count data were normalized using the SCTransform function implemented in Seurat (note that this normalized expression profile was only used for feature preprocessing). Following normalization, the top 3000 highly variable genes were selected. For ATAC-seq data, peaks from both multiome and scATAC-seq data were merged. Combined peaks were created using the reduce function in Signac, followed by re-quantification of peak accessibility using the merge function in Signac. Peaks were then filtered based on their proximity to TSS and correlation with gene expression in multiome data. First, the LinkPeaks function implemented in Signac was applied around TSS regions (±50kb) to identify peaks associated with gene expression. Genes exhibiting a significant correlation (*P* < .05) with at least 10 peaks were identified as DORC genes, and these genes were added to the list of RNA-seq genes. Next, the LinkPeaks function was applied within ±500kb of the TSS, and peaks showing high absolute correlation values (>0.01) with any of the RNA-seq genes were selected as features. The resulting filtered count matrix of gene expression and chromatin accessibility was used as the input for mmVelo.

## 3 Results

### 3.1 mmVelo model

**Figure 1:**
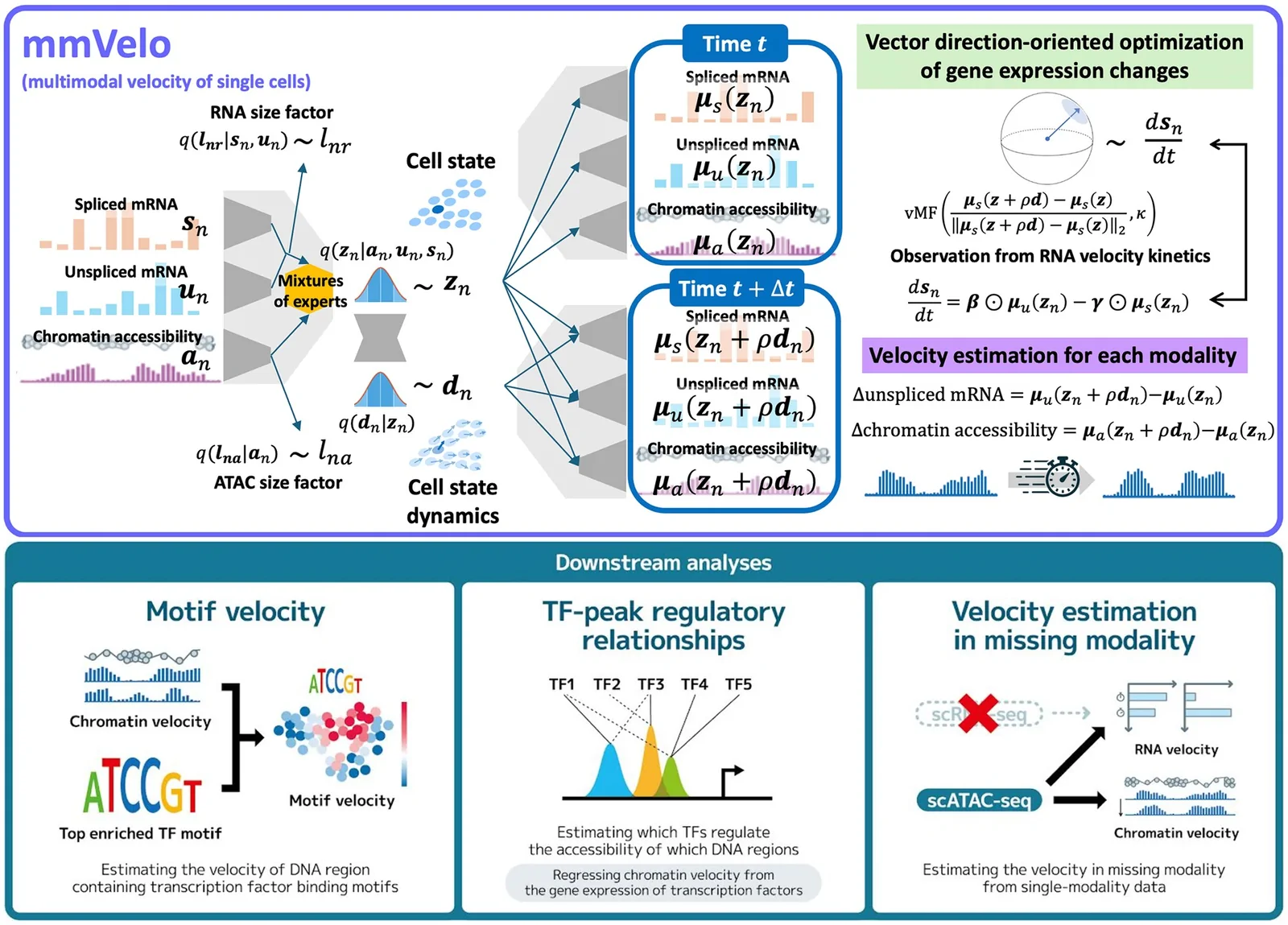
Overview of mmVelo. mmVelo uses single-cell multiomics data as input data and outputs the velocity for each modality. It estimates cell state-dependent dynamics within the cell state space and estimates velocity by mapping these dynamics to the space of each modality. The estimation of cell state-dependent dynamics is conducted based on the assumption of splicing kinetics. Downstream analyses include estimating changes in TF motif accessibility rates, identifying TFs that control chromatin accessibility at specific loci, and estimating velocity in a missing modality using single-modality measurement data as input data.

mmVelo uses single-cell multiomics data as input data and outputs the dynamics of each modality (Fig. 1). Let $a_{n}$, $u_{n}$, and $s_{n}$ represent chromatin accessibility, unspliced mRNA expression, and spliced mRNA expression in the cell *n*, respectively. Using the encoders for each modality, mmVelo infers the distribution $q(z_{n}|a_{n},u_{n},s_{n})$, representing the cell state. This distribution is expressed as a mixture of experts from the multivariate Gaussian distributions $q(z_{n}|a_{n})$ and $q(z_{n}|u_{n},s_{n})$ that are inferred from each modality. Samples from the cell state distribution $q(z_{n}|a_{n},u_{n},s_{n})$ are then used as input data for a decoder for each modality, outputting the probability distributions $p(a_{n}|z_{n})$, $p(u_{n}|z_{n})$, and $p(s_{n}|z_{n})$ corresponding to each modality. Next, mmVelo infers the distribution $q(d_{n}|z_{n})$ representing the transition vector $d_{n}$ of the cell state over a small-time interval, using samples from the cell state distribution $q(z_{n}|a_{n},u_{n},s_{n})$. The transition of the cell state over a short time interval was estimated to be consistent with the RNA velocity $ds_{n}/dt$, which represents the changes in gene expression described based on splicing kinetics. Finally, mmVelo estimates the temporal changes in each modality by mapping the current cell states ($z_{n}$) and the cell states after the small-time interval ($z_{n}+\rho d_{n}$) using the decoder of each modality.

Single-cell multiomics data is difficult to analyze owing to its inherent sparsity and noise compared with single-modality data. This complexity can impede accurate estimation of the differentiation directions in traditional RNA velocity models (Ma *et al.* 2020c, Li *et al.* 2023). To address this issue, mmVelo integrates multimodal profiles to define cell states, allowing for a more robust estimation of cell states and their neighboring relationships (Gayoso *et al.* 2021, Minoura *et al.* 2021). Furthermore, by modeling RNA velocity along cell state transitions within a low-dimensional manifold, mmVelo accounts for gene–gene coherence, which is not considered in traditional methods that independently solve differential equations for each gene (Gorin *et al.* 2022, Nagaharu *et al.* 2022, Lederer *et al.* 2024). To capture the overall direction of gene expression changes, mmVelo employs a von Mises–Fisher distribution for the probability distribution of RNA velocity.

A unique feature of mmVelo is its ability to model chromatin accessibility at the peak level, enabling the estimation of chromatin velocity at a single-peak resolution. This capability allows for dynamic analysis and inference of regulatory relationships at this level. Additionally, by leveraging the temporal changes at the single-peak resolution, mmVelo estimates the dynamics of TF binding motifs at the single-cell level. Another distinctive aspect of mmVelo is its use of multimodal representation learning, which facilitates the prediction of velocities in missing modalities using single-modality data, such as inferring chromatin velocity from scRNA-seq data. Detailed model descriptions and downstream analyses are presented in the Methods section.

### 3.2 Robustness of mmVelo to latent dimensionality and smoothing

We assessed the robustness of mmVelo to the choice of latent dimensionality and the use of smoothing (Fig. S5, available as supplementary data at *Bioinformatics* online). We performed an ablation analysis on the embryonic mouse brain and SHARE-seq mouse skin hair follicle datasets using latent dimensions of 5, 10, 30, and 50, and additionally evaluated a no-smoothing condition at latent dimension 10. For each setting, we computed the VCS and visualized the inferred chromatin velocity field using streamline plots (Methods).

In the embryonic mouse brain dataset, the VCS values were broadly comparable across latent dimensionalities, although higher-dimensional settings (*z* = 30 and *z* = 50) tended to show slightly higher scores overall (Fig. S5a, available as supplementary data at *Bioinformatics* online). However, the streamline plots indicated that the default setting (*z* = 10) better avoided weak reverse flow near terminal populations, whereas the other dimensionalities and the no-smoothing setting showed slight backflow in terminal regions (Fig. S5b, available as supplementary data at *Bioinformatics* online). In the SHARE-seq mouse skin hair follicle dataset, the *z* = 10 setting yielded the highest VCS, and smoothing improved performance relative to the no-smoothing condition(Fig. S5c, available as supplementary data at *Bioinformatics* online). Moreover, the streamline plot obtained with *z* = 10 and smoothing was the most consistent with the known biological direction of differentiation (Fig. S5d, available as supplementary data at *Bioinformatics* online).

Because the hair follicle dataset contains multiple lineages, the VCS in this dataset should be interpreted with some caution, as it was computed using a single pseudotime axis. Nevertheless, taken together, these analyses indicate that the default configuration (*z* = 10 with smoothing) provides stable performance across datasets and offers a practical balance between robustness and biological fidelity.

### 3.3 mmVelo accurately estimates chromatin accessibility dynamics in embryonic mouse brain

**Figure 2:**
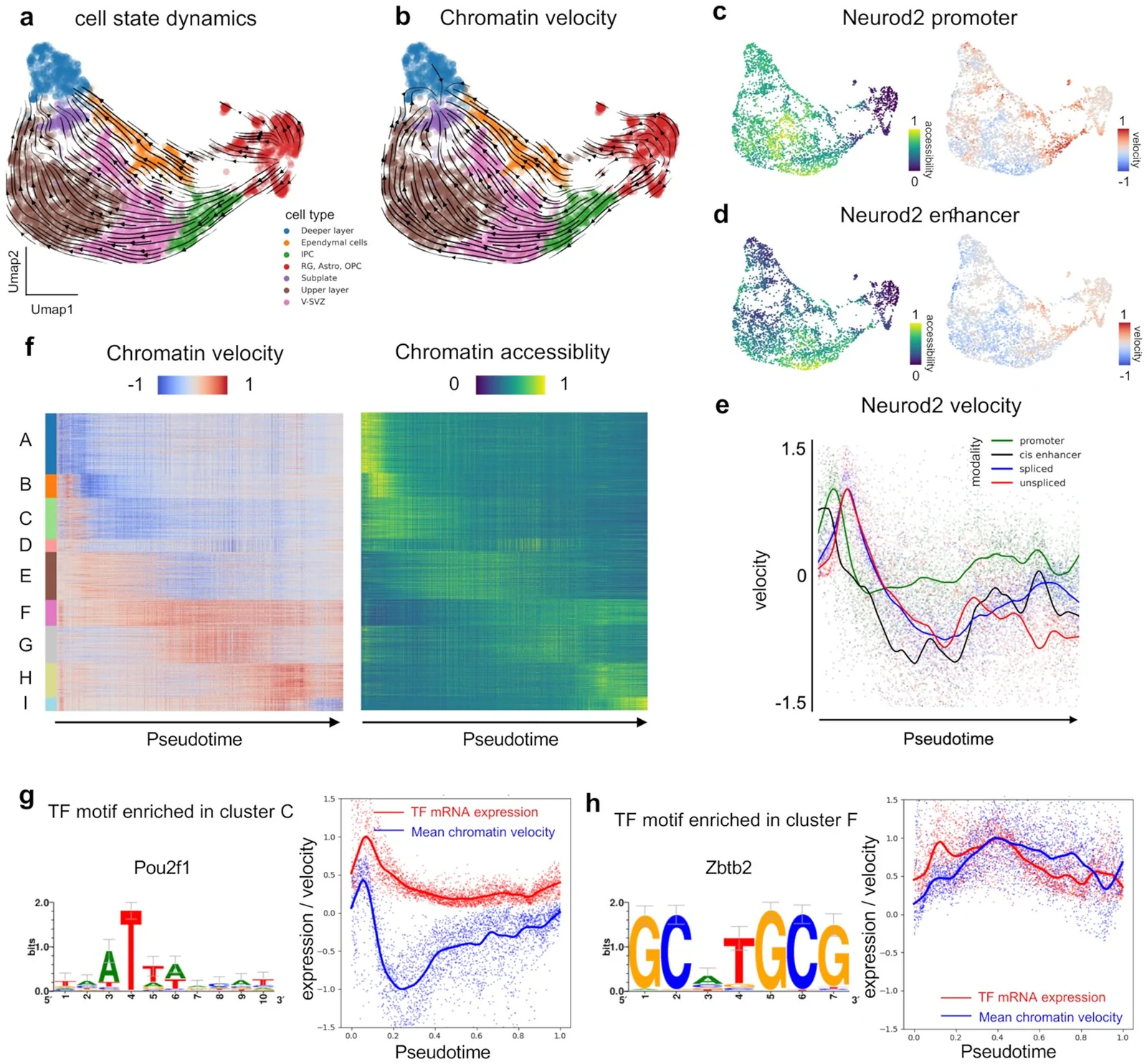
mmVelo accurately estimates chromatin accessibility dynamics in embryonic mouse brain. (a-b) UMAP coordinates with stream plots of cell state dynamics (a) and chromatin velocity (b) from mmVelo. IPC, intermediate progenitor cells; RG, radial glia; Astro, astrocytes; OPC, oligodendrocyte progenitor cells; V-SVZ, ventricular-subventricular zone. (c-d) UMAP plots colored by chromatin accessibility (left) and chromatin velocity (right) of the *Neurod2* promoter region (chr11:98329299–98330151) (c) and enhancer region (chr11:98320243–98320844) (d). (e) Dynamics of *Neurod2* in each modality along the pseudotime axis. (f) Heatmaps of chromatin velocity (left) and chromatin accessibility (right) along pseudotime axis. Peaks with similar chromatin velocity patterns were classified into nine peak clusters. (g-h) Trends in average chromatin velocity within clusters and gene expression corresponding to TF motifs enriched in the cluster. Motifs enriched among the peaks in cluster C (g) and cluster F (h) are shown.

We first applied mmVelo to 10× multiome data obtained from the embryonic mouse brain on embryonic day 18 (E18). Based on the inferred cell state dynamics and chromatin velocity, the velocity vectors accurately captured the known developmental trajectory of the mammalian cortex (Fig. 2a and b). Specifically, the radial glia cells in the outer subventricular zone are the progenitors of neurons, astrocytes and oligodendrocytes (Merkle *et al.* 2004, Hansen *et al.* 2010, Pollen *et al.* 2015). Radial glia cells divide into intermediate progenitor cells that give rise to mature excitatory neurons across various cortical layers (Mayer *et al.* 2019). The formation of cortical layers occurs through a process in which neurons migrate in an inside-out manner, with newer cells ascending to the upper layers and older cells remaining in the deeper strata(Nadarajah and Parnavelas 2002).

One of the unique aspects of mmVelo is its ability to estimate chromatin velocity at a single-peak resolution. We present the estimated accessibility changes in the promoter and enhancer regions of *Neurod2* (Fig. 2c and d). The chromatin velocity estimated using mmVelo accurately captured the changes in accessibility along the trajectory of cell differentiation. While promoter regions only undergo upregulation of accessibility during the early stages of differentiation, enhancer regions also undergo subsequent downregulation. *Neurod2* is a TF belonging to the basic Helix-loop-Helix superfamily, and its transient expression confers a neuronal fate to stem cells (Lin *et al.* 2004, Cherry *et al.* 2011). These results demonstrated that mmVelo accurately infers the dynamics of chromatin accessibility at the single-peak resolution.

Modelling cell state dynamics on multimodal cell states allows mmVelo to simultaneously estimate the velocity of multiple modalities for each cell, enabling to examine differences in temporal regulation across modalities. To verify this hypothesis, we investigated the velocity of *Neurod2* along the pseudotime axis for each modality in the excitatory neuron lineage (Fig. 2e). We confirmed a trend in which changes in the enhancer region occurred first, followed by changes in the promoter region, and finally, changes in gene expression. This is consistent with the findings of Ma *et al.* (2020c), who reported that the regulatory regions of lineage-determining genes were accessible prior to the onset of gene expression. This result highlights that mmVelo captures not only consistent developmental directionality across modalities, but also biologically meaningful time lags between regulatory and transcriptional events.

Next, we hypothesized that the rate of change in chromatin accessibility reflects the effects of regulation more directly than chromatin accessibility itself, and that chromatin regions affected by common regulators exhibit synchronous accessibility dynamics. To test this hypothesis, we performed Leiden clustering (Traag *et al.* 2019) on peaks based on chromatin velocity and identified nine peak clusters (Fig. 2f). These clusters are defined in the space of peaks rather than cells, and therefore represent groups of regulatory regions with similar temporal accessibility changes. The resulting clusters revealed distinct patterns of accessibility dynamics along pseudotime, capturing chromatin regions that changed synchronously during development. To facilitate interpretation of these peak clusters in relation to cell-state structure, we computed the average chromatin velocity of peaks within each cluster and projected it onto the cellular UMAP (Fig. S6, available as supplementary data at *Bioinformatics* online). This analysis showed that each peak cluster was preferentially associated with specific regions or phases of the differentiation manifold, supporting the biological relevance of the identified peak clusters.

Similar change patterns observed within each cluster suggest the presence of common regulatory factors. To identify the putative TFs that govern these accessibility changes, we conducted motif enrichment analysis for each peak cluster using pycisTarget. Next, we explored the relationships between the expression patterns of TFs (corresponding to the top-enriched motifs in each cluster) and the average chromatin velocity of the peaks belonging to the cluster (Fig. 2g and h). Notably, *Zbtb2* in cluster F strongly correlated with chromatin velocity and TF expression, suggesting that it plays a role in regulating chromatin accessibility. *Zbtb2* recruits chromatin remodelers and histone chaperones to control differentiation, highlighting its regulatory role in chromatin accessibility (Olivieri *et al.* 2021). These findings demonstrate that mmVelo estimates of temporal changes in chromatin accessibility facilitate the identification of factors that govern the chromatin landscape.

### 3.4 mmVelo reveals the dynamics of TF binding motifs in mouse hair follicle development

**Figure 3:**
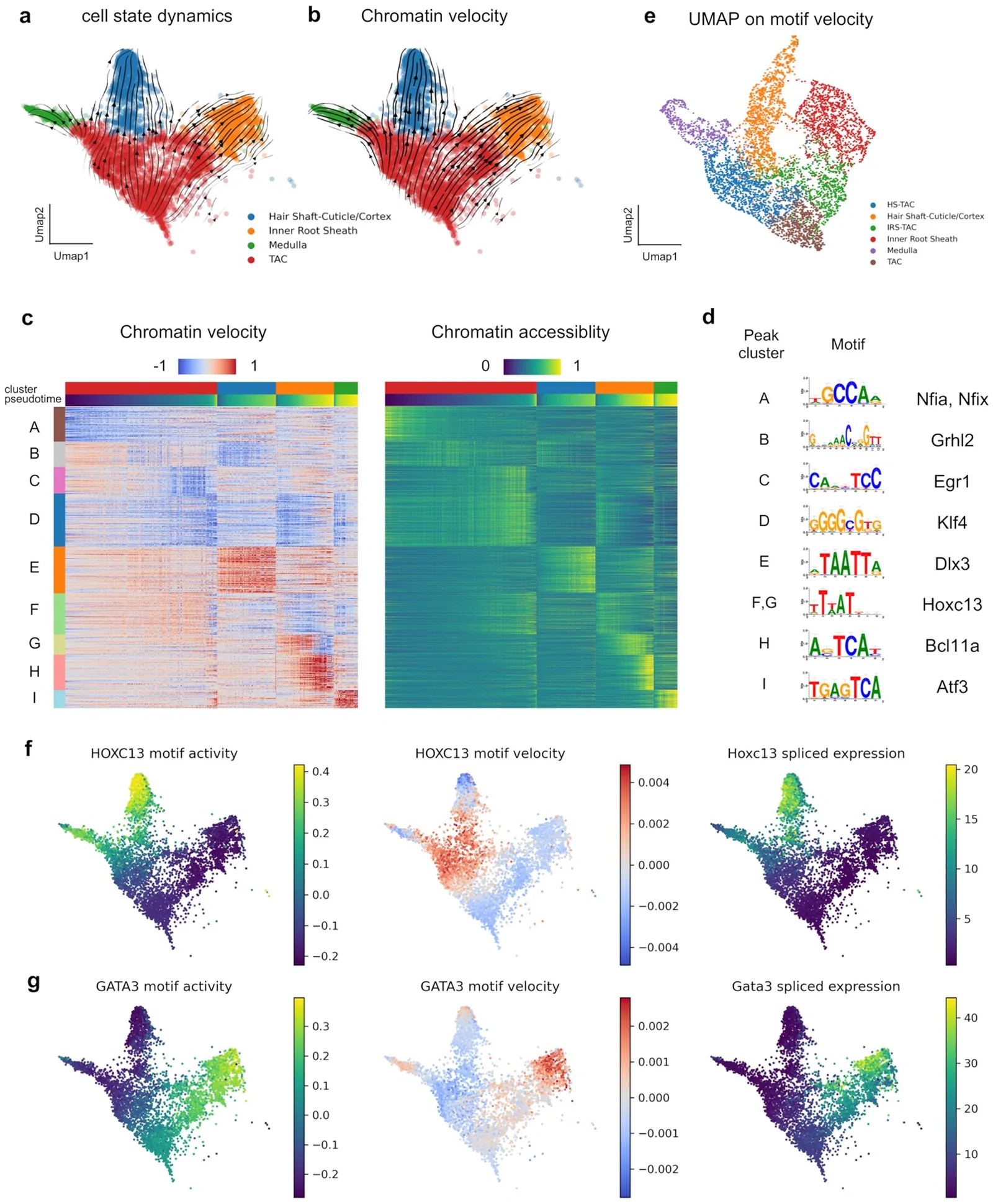
mmVelo reveals the dynamics of TF binding motifs in mouse hair follicle development. (a-b) UMAP coordinates with stream plots of cell state dynamics (a) and chromatin velocity (b) from mmVelo. TAC, transit-amplifying cells. (c) Heatmaps of chromatin velocity (left) and chromatin accessibility (right) along pseudotime axis. Peaks with high similarity in chromatin velocity are classified into nine clusters. (d) Top enriched TF motifs in each peak cluster. (e) UMAP representations of the motif velocity. (f-g) UMAP plots colored by motif activity (left), motif velocity (middle), and spliced mRNA expression (right) of *Hoxc13* (f) and *Gata3* (g).

Next, we applied mmVelo to mouse skin hair follicle differentiation processes assayed using SHARE-seq (Ma *et al.* 2020c). The inferred cell state dynamics and chromatin velocity accurately captured the direction of differentiation, as reported in the initial study (Fig. 3a and b). This progression involves the differentiation of transit-amplifying cells into the internal root sheath, medulla, and cuticle/cortex (Zhang *et al.* 2016, Zhang and Hsu 2017). Furthermore, benchmark analyses using hair shaft-cuticle/cortex lineage cells demonstrated that mmVelo consistently estimated chromatin velocity and RNA velocity with higher accuracy than existing models, including MultiVelo (Li *et al.* 2023) and scVelo (Bergen *et al.* 2020; Fig. S3j–m, available as supplementary data at *Bioinformatics* online).

To identify TFs that regulate chromatin accessibility, we performed Leiden clustering on peaks using chromatin velocity estimated by mmVelo and identified nine distinct peak clusters (Fig. 3c). Using pycisTarget (Bravo González-Blas *et al.* 2023), we conducted a motif enrichment analysis for each cluster of peaks and confirmed the enrichment of TF binding motifs (Fig. 3d). Among the enriched motifs, *Nfix*, an NFI TF involved in maintaining stem cell identity by preserving chromatin accessibility (Adam *et al.* 2020), and *Hoxc13*, which is essential for proper hair follicle differentiation (Jave-Suarez *et al.* 2002) were identified. These findings suggest that the chromatin velocity inferred by mmVelo facilitates the elucidation of dynamic regulation and concurrent changes in chromatin accessibility.

To further elucidate the dynamic regulation of TF binding motifs associated with differentiation, we computed the accessibility dynamics of these motifs, which we refer to as motif velocity (Methods). Dimensional reduction of the motif velocity using UMAP recapitulated the continuous transitional changes in cell populations associated with hair follicle differentiation (Fig. 3e), indicating that the estimated motif velocity captured the biological variations in cell states. In particular, the motif velocities of the TFs *Hoxc13* and *Gata3*, which are essential for differentiation into the hair shaft and internal root sheath lineages (Kaufman *et al.* 2003), respectively, accurately captured the changes in motif activity that occur during differentiation (Fig. 3f and g). Furthermore, these changes were concurrent with the gene expression patterns, illustrating the synchronized regulation of the cistrome with gene expression during the differentiation process. These findings demonstrate that in addition to estimating the dynamics of chromatin accessibility, the mmVelo framework enables the estimation of cistrome dynamics.

### 3.5 Posterior velocity variability reveals modality-specific uncertainty patterns

**Figure 4:**
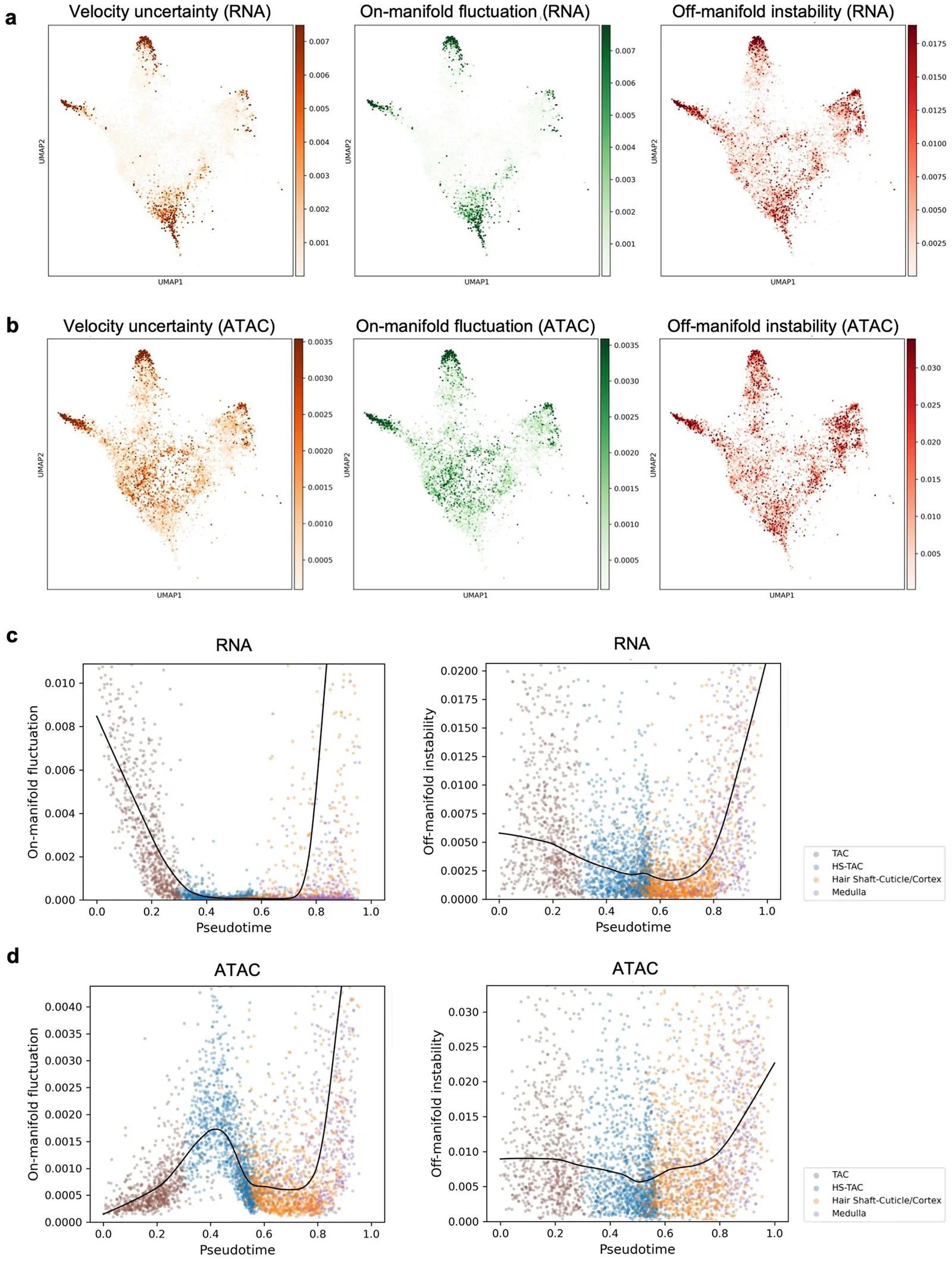
Posterior velocity variability and its decomposition in mouse hair follicle differentiation. (a-b) UMAP coordinates colored by velocity uncertainty (left), on-manifold fluctuation (middle), and off-manifold instability (right) for RNA (a) and ATAC (b). For each cell, *S* = 100 posterior samples of the transition vector were drawn and mapped to each modality to obtain velocity samples. Each metric is defined as the across-sample variance of the cosine similarity between individual velocity samples and the sample-mean velocity. Because cosine similarity is bounded in [–1, 1], values are small in absolute terms: values near 0 indicate consistent velocity directions (high confidence) and larger values indicate greater directional spread (lower confidence). Velocity uncertainty (left) is computed from the full velocity vector, while on-manifold fluctuation (middle) and off-manifold instability (right) are computed from the velocity components lying within and orthogonal to the local tangent space, respectively, estimated in each modality-specific low-dimensional embedding (Methods). Color scales are capped at the 95th percentile. (c, d) Trends in on-manifold fluctuation (left) and off-manifold instability (right) for RNA (c) and ATAC (d); the metrics are as defined in (a-b). Dots are colored by cell type.

We next asked whether the posterior variability of mmVelo could provide interpretable information about the confidence and structure of inferred dynamics. Using the mouse hair follicle dataset, we quantified posterior velocity variability for RNA and chromatin modalities by repeated sampling of the inferred transition vector, followed by decomposition into manifold-aligned and off-manifold components (Fig 4a and b; Methods).

To visualize these patterns along differentiation, we focused on the hair shaft and medulla lineages and summarized the variability along pseudotime (Fig. 4c and d). The off-manifold component increased toward terminal populations in both modalities, consistent with reduced local support near sparse boundary regions of the cell-state manifold. This pattern is consistent with the interpretation that decoder-based predictions become more sensitive to extrapolation in such regions, and therefore that elevated off-manifold instability may serve as a surrogate for lower-confidence velocity estimates.

In contrast, the manifold-aligned component showed modality-specific behavior. The manifold-aligned component of chromatin velocity variability was elevated near lineage branching regions, a pattern that was not observed in RNA velocity (Fig. 4d). This modality-specific signature suggests that chromatin-level fluctuations may precede or accompany fate decisions, consistent with the view that regulatory regions become accessible prior to transcriptional commitment. Elevated variability was also observed near terminal populations, which we interpret cautiously as a combination of possible structured fluctuation and residual numerical instability in sparse boundary regions.

Together, these results indicate that posterior velocity variability in mmVelo contains interpretable structure beyond a single scalar uncertainty score: the off-manifold component highlights regions of potentially unstable prediction, whereas the manifold-aligned component may capture modality-specific biological variability along differentiation.

### 3.6 Chromatin velocity enables inference of putative regulatory TFs for each regulatory region

In the previous section, we showed that considering chromatin velocity alongside TF gene expression allows for the exploration of factors regulating chromatin accessibility. Based on this concept, we hypothesized that chromatin velocity utilization would enable the identification of TFs involved in regulating individual peaks of chromatin accessibility. To demonstrate this, we estimated the regulatory relationships between TFs and chromatin accessibility peaks.

**Figure 5:**
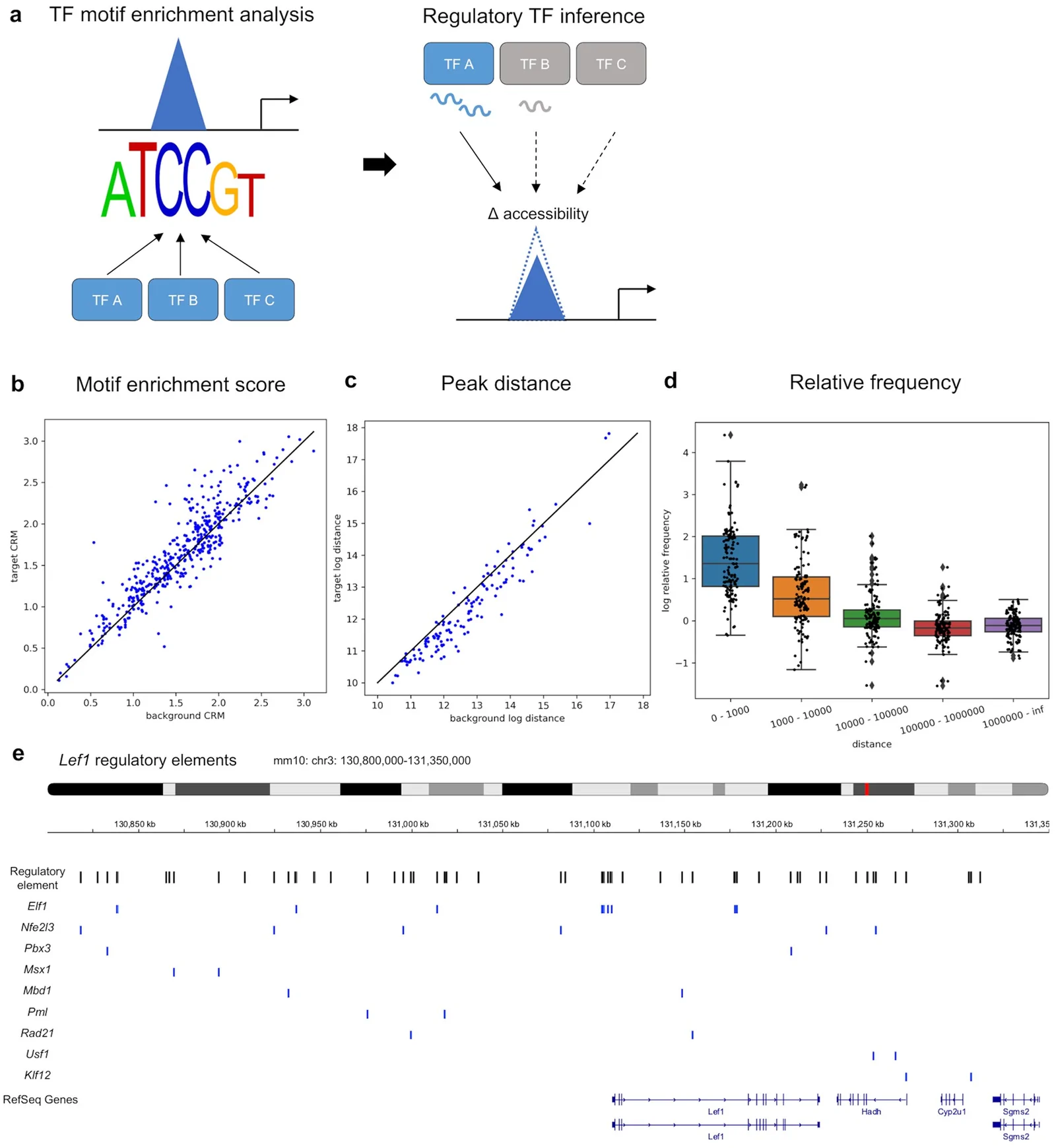
Chromatin accessibility dynamics enables inference of putative regulatory TFs for each regulatory region. (a) Illustration of regulatory relationship inference between TFs and chromatin accessibility peaks. (b) Scatter plot representing the CRM scores of TF motifs for TF-targeted peaks (*y*-axis) and non-targeted peaks within the same cluster (*x*-axis). Each point corresponds to a TF motif. (c) Scatter plot representing the mean genomic distances (in base pairs) between TF-targeted peaks (*y*-axis) and non-targeted peaks (*x*-axis) on the same chromosome. (d) Distributions of log-relative frequencies of distances between peaks among targeted and non-targeted peaks. (e) The inferred regulatory relationships between TF and peaks within the *Lef1* gene regulatory elements (chr3: 130 800 000–131 350 000).

We inferred the regulatory relationships between TFs and chromatin accessibility peaks using a three-step process (Fig. 5a, Methods), grounded in the SCENIC + framework (Bravo González-Blas *et al.* 2023). Initially, motif enrichment analysis for each cluster of peaks was conducted to identify the candidate TFs involved in regulation. Subsequently, we quantified the importance scores of candidate regulatory TFs in the regression task of chromatin velocity based on their mRNA expression. Finally, the regulatory relationships between TFs and chromatin accessibility peaks were estimated based on importance scores. In our experiment, we estimated the regulatory relationships between 101 644 TF-peak pairs.

To validate the accuracy of the inferred regulatory relationships, we compared the degree-of-motif enrichment between TF-regulated and non-regulated peak sets within the same peak clusters. The average likelihood of occurrence of relevant motifs (CRM scores; Cis regulatory modules scores) in the TF-regulated peak sets was higher than that in the non-regulated peak sets (Fig. 5b) (*P* < .05, Wilcoxon rank-sum test). This suggests that the extracted regulated peaks contained sequences that were more likely bound by the respective TFs. These results indirectly corroborated the accuracy of the estimated regulatory relationships.

Topologically associated domains (TADs) are regions within the genome characterized by self-interacting sequences. The DNA sequences within each TAD interact with each other at a higher frequency than those outside the domain (Pombo and Dillon 2015, Tena and Santos-Pereira 2021). Inspired by this concept, we hypothesized that TF-regulated chromatin accessibility tends to be concentrated in regions that are genomically proximate. To test this hypothesis, we compared the distances between TF-regulated peaks to the distances between random peaks within the same chromosome. Consistent with our hypothesis, the distances between the regulated peaks were shorter than those between the random peaks (Fig. 5c;  *P* < .01, Wilcoxon rank-sum test). To determine the extent to which each TF’s regulated peak is concentrated within a certain distance range, we compared the distribution of the genomic distance between the peaks regulated by a common TF and those between randomly selected peaks. The results showed the concentration of TF-regulated peaks within a range of up to 100 kb (Fig. 5d). These results suggest that the regulation of chromatin accessibility by TFs is concentrated within localized regions of the chromosome.

As an example of the inferred regulatory relationships, the interactions between TFs and peaks in the *Lef1* regulatory elements are shown in (Fig. 5e). For each peak, we depicted the TF predicted to have the most significant positive regulatory effect. *Elf1* and *Nfe2l3* were identified as the TFs that regulate the largest number of regulatory sequences. Notably, *Elf1* was predominantly localized near the coding regions of *Lef1*, consistent with its role as a transcription elongation factor that binds to these regions (Prather *et al.* 2005). These results demonstrate that chromatin velocity utilization facilitates regulatory relationship inference.

### 3.7 mmVelo estimates velocity in missing modalities

**Figure 6:**
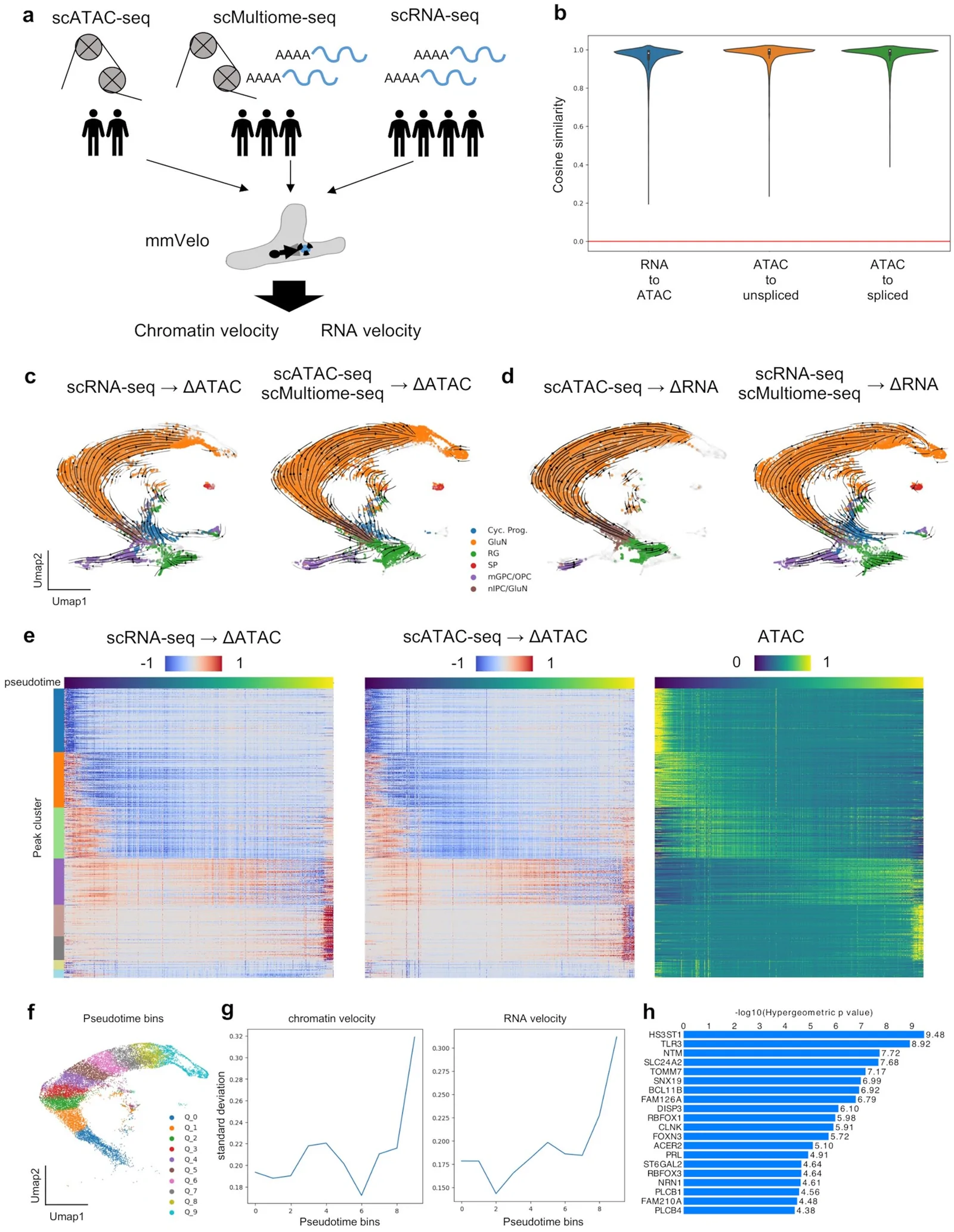
mmVelo estimates velocity in missing modalities. (a) Diagram of dynamics estimation experiment in missing modality. (b) Violin plot showing the average cosine similarity between the velocity estimated in missing modality from single-modality data and the velocity in the 10 nearest neighbor cells from multiome data. (c) UMAP coordinates with stream plot of chromatin velocity inferred from scRNA-seq (left) and from multiome and scATAC-seq (right). Cells measured in the queried modality are shown in color, whereas cells without that modality are shown in gray. Cyc.Prog., cycling progenitors; GluN, glutamatergic neuron; RG, radial glia; SP, subplate; mGPC/OPC, multipotent glial progenitor cell/oligodendrocyte progenitor cell; nIPC, neuronal intermediate progenitor cell. (d) UMAP coordinates with stream plot of RNA velocity inferred from scATAC-seq (left) and from multiome and scRNA-seq (right). (e) Heatmaps of chromatin velocity inferred from scRNA-seq (left), scATAC-seq (middle), and chromatin accessibility (right) along pseudotime axis in GluN lineage. (f) UMAP coordinates of excitatory neuron lineage cells colored by pseudotime bins. Only cells included in the GluN lineage analysis are shown; cells outside this lineage are not displayed. (g) Trends in the average inter-sample variability of chromatin velocity (left) and RNA velocity (right) within each pseudotime bin. (h) Genes enriched in the top 10% of peaks showing high inter-sample variability in the Q4 bin, identified by hypergeometric testing.

Although single-cell multiomics technologies are advancing, these methods are still expensive and less universally applicable than single-modality measurements such as scRNA-seq and scATAC-seq. Therefore, the ability to predict missing modalities from single-modality data provides more profound insights into biological systems in a cost-effective manner. Although several methods can estimate profiles in missing modalities (Minoura *et al.* 2021, Ashuach *et al.* 2023), no current method can estimate the dynamics of missing modalities. To address this, we developed a method to jointly estimate RNA and chromatin velocities from singleome data via scRNA-seq, scATAC-seq, and multiome data integration into the mmVelo framework (Fig. 6a, Methods).

As a proof-of-concept, we generated a simulated dataset, with a missing modality, from a 10× multiome dataset of human cortical development (Trevino *et al.* 2021) and evaluated the estimated velocity of the missing modality. Multiomics data derived from three samples were manipulated such that one sample had an artificially induced missing RNA modality and the other had a missing ATAC modality. We applied mmVelo to this simulated dataset to estimate the velocity of the missing modality. The velocities inferred for the missing modality by mmVelo were consistent with the mean velocities of 10 neighboring multiome cells (Fig. 6b), demonstrating that the model is capable of accurately estimating the dynamics of missing modalities.

Next, we applied mmVelo to a dataset comprising scRNA-seq, scATAC-seq, and 10× multiome data obtained from the human cortical development process, all measured at the same PCW21. Vector fields based on RNA velocity estimated from scATAC-seq and chromatin velocity estimated from scRNA-seq correctly captured the known biological directionality of differentiation (Fig. 6c and d). Furthermore, the dynamics estimated for the missing modalities in the excitatory neuron lineage captured the observed temporal changes in accessibility and gene expression (Fig. 6e). These results demonstrate that mmVelo accurately predicts temporal changes in missing modalities.

The incorporation of singleome data facilitates an increase in the number of samples simultaneously analyzed. In our experiment, integrating multiome data from three individuals with scRNA-seq and/or scATAC-seq data from four additional individuals enabled statistical inferences using a total of seven specimens. Leveraging this, we analyzed the inter-individual variability in dynamics, focusing specifically on the lineage of excitatory neurons, to identify the stage of cell differentiation at which the variability in dynamics becomes pronounced. To quantify the variability in chromatin and RNA velocity among individuals, we divided the excitatory neuron lineage cells into 10 bins based on pseudotime and computed the variance in the mean velocity for each sample within each bin (Fig. 6f, Methods). Notably, the timing of inter-sample variability differed between modalities: larger fluctuations emerged first in chromatin accessibility and were followed by changes in RNA velocity (Fig. 6g). This modality-specific temporal offset suggests that regulatory variation may arise before transcriptional divergence becomes apparent. To investigate the functional significance of the top fluctuating peaks, hypergeometric tests were conducted on these peak-associated genes using GREAT (McLean *et al.* 2010). Among the top fluctuating genes, several were associated with the cerebral cortex development or with neurological disorders (Fig. 6h;  Fogel *et al.* 2012, Miyamoto *et al.* 2014, Konířová *et al.* 2017, Lennon *et al.* 2017, Witoelar *et al.* 2018, Ma *et al.* 2020b, Zhang *et al.* 2023). This suggests that certain regulatory mechanisms in neuronal differentiation exhibit significant inter-individual variability, potentially reflecting underlying biological or environmental differences. These results demonstrate the utility of integrating single-modality data for velocity analysis to gain biological insights, highlighting the potential of mmVelo to enhance our understanding of the complex dynamics of cellular processes among different individuals.

## 4 Discussion

RNA velocity analysis is a powerful method for uncovering the dynamics of gene expression within cells. However, existing approaches are limited to RNA modalities, and estimating velocity for other modalities, such as chromatin accessibility, remains a major challenge. To address this gap, we introduce mmVelo, a framework based on a mixture of experts VAE designed to infer multimodal cell states and their dynamics across multiple modalities.

A key innovation of mmVelo is its ability to model chromatin velocity at a single-peak level, which enables the detailed analyses of time lags between regulatory regions of the same gene, TF motif dynamics, and regulatory relationships with a single-peak resolution. Our experiments revealed how the promoter and enhancer regions of *Neurod2* progressively become accessible over time. By analyzing individual peak dynamics, we inferred the behavior of aggregated cistromes. This was accomplished by extending the motif deviation score from chromVAR to calculate motif velocity (Schep *et al.* 2017), allowing us to quantify the rate of change in lineage-determining TF motifs critical for mouse hair follicle differentiation. Additionally, we extended the SCENIC + framework to regress chromatin velocity from TF mRNA expression (Bravo González-Blas *et al.* 2023), enabling the identification of TFs that regulate peak accessibility. These advancements surpass existing methods, such as MultiVelo (Li *et al.* 2023), which aggregate chromatin accessibility at the gene level, providing high-resolution dynamic analyses and deeper insights into regulatory mechanisms across multiple modalities.

Beyond demonstrating concordance across modalities, mmVelo also revealed biologically informative differences in their temporal behavior. In the *Neurod2* analysis, enhancer accessibility changed first, followed by promoter accessibility and then RNA dynamics, indicating a clear time lag between regulatory and transcriptional events. This pattern is consistent with the SHARE-seq study (Ma *et al.* 2020c), which showed that regulatory regions of lineage-determining genes become accessible prior to the onset of gene expression, supporting a model of chromatin-mediated lineage priming. Viewed in this context, our results suggest that multimodal velocity analysis can capture not only the overall direction of differentiation, but also the temporal ordering by which chromatin remodeling precedes and potentially conditions later transcriptional commitment. In addition, the earlier emergence of chromatin-level variability relative to RNA-level variability, together with the chromatin-specific increase in manifold-aligned fluctuation near lineage branching regions, further supports the idea that regulatory changes may precede or accompany fate decisions. These observations highlight that differences between modalities are themselves biologically informative and constitute an important advantage of multimodal velocity analysis.

In addition, mmVelo’s multimodal representation learning, built on a mixture of experts VAE framework, allows for the prediction of velocities in missing modalities. For example, using a human cortical development dataset, we demonstrated that both RNA velocity and chromatin velocity can be inferred from scATAC-seq data. While existing models such as scMM (Minoura *et al.* 2021) and MultiVI (Ashuach *et al.* 2023) predict missing modality profiles, they do not estimate the dynamics of these modalities. By applying mmVelo to large single-modality datasets and bridging them with multiomics datasets, researchers can expand the scope of existing data analyses, uncovering novel insights into cellular dynamics and their regulation.

A promising future direction is to integrate mmVelo-based velocity estimation with various mathematical models beyond RNA velocity. For instance, models such as MultiVelo (Li *et al.* 2023), which is based on chromatin-gene regulatory relationships, or scKINETICS (Burdziak *et al.* 2023), which is based on gene regulatory networks, can be employed. This integration could offer a more detailed exploration of regulatory relationships within and between modalities and enable dynamic estimation in a broader range of systems, including those in which RNA velocity applications are challenging (Bergen *et al.* 2021). Additionally, combining this approach with fate mapping tools, such as CellRank (Lange *et al.* 2022, Weiler *et al.* 2024), could help to identify regulatory regions that contribute to cell lineage decisions.

Another important direction is to extend mmVelo to incorporate condition-specific dynamics and identify the contributing factors. Although our model currently assumes that all cells follow the same dynamics, it is plausible that different dynamics exist under specific conditions, such as under different diseases. While studies have been conducted on condition-specific dynamics estimation at the gene expression level (Kojima *et al.* 2024), research extending such estimations across multiple modalities is yet to be conducted. By elucidating the dynamics across modalities and their contributing factors under specific conditions, we can advance our understanding of specific disease mechanisms and drug responses across various molecular layers.

In summary, mmVelo enables the estimation of the dynamics of each modality from single-cell multiomics data. We anticipate that mmVelo will maximize the potential of multiomics data, providing insights into the regulatory relationships across various molecular layers in diverse biological systems, including development, differentiation, and disease.

## 4.1 Limitations of the study

mmVelo estimation of cell state dynamics is based on RNA velocity; therefore, it cannot be applied to biological systems where RNA velocity-based estimation of the directionality of cell state changes is difficult (Bergen *et al.* 2021). Nevertheless, it is crucial to note that the dynamics estimation method proposed in this study can be applied to systems that previously challenged traditional RNA velocity methods (e.g. see the application of RNA velocity to hair follicle differentiation in Ma *et al.* 2020c). Another limitation of our model is the use of smoothed profiles to address the sparsity of observations (Gorin *et al.* 2022, Zheng *et al.* 2023), along with the assumption of splicing kinetics based on a single kinetic rate (Bergen *et al.* 2021). Expanding the model to include count modeling (Gorin and Pachter 2022, Lederer *et al.* 2024) and estimating the dynamics based on variable kinetic rates (Li *et al.* 2024, Mizukoshi *et al.* 2024) are future research directions.

## Supplementary Material

btag652_Supplementary_Data

## Data Availability

The 10× embryonic mouse brain dataset was downloaded from the 10× website at https://www.10xgenomics.com/datasets/fresh-embryonic-e-18-mouse-brain-5-k-1-standard-2-0-0. The SHARE-seq mouse skin dataset (Ma *et al.* 2020c) was downloaded from GEO (GSE140203). The 10× human cortical development dataset was downloaded from GEO (GSE162170). The full source code is publicly available at https://github.com/nomuhyooon/mmVelo, and an archived version corresponding to this study is available at https://doi.org/10.5281/zenodo.20103609. Any additional information required to reanalyze the data reported in this paper is available from the lead contact upon request.
