# Supplementary material for: mmVelo: a deep generative model for estimating cell state-dependent dynamics across multiple modalities
Source: Bioinformatics. 2026 Aug 31;42(9):btag652. doi: 10.1093/bioinformatics/btag652 (PMC13585172; doi:10.1093/bioinformatics/btag652)
Supplement: Supplementary file 1 [file btag652_supplementary_data.pdf]

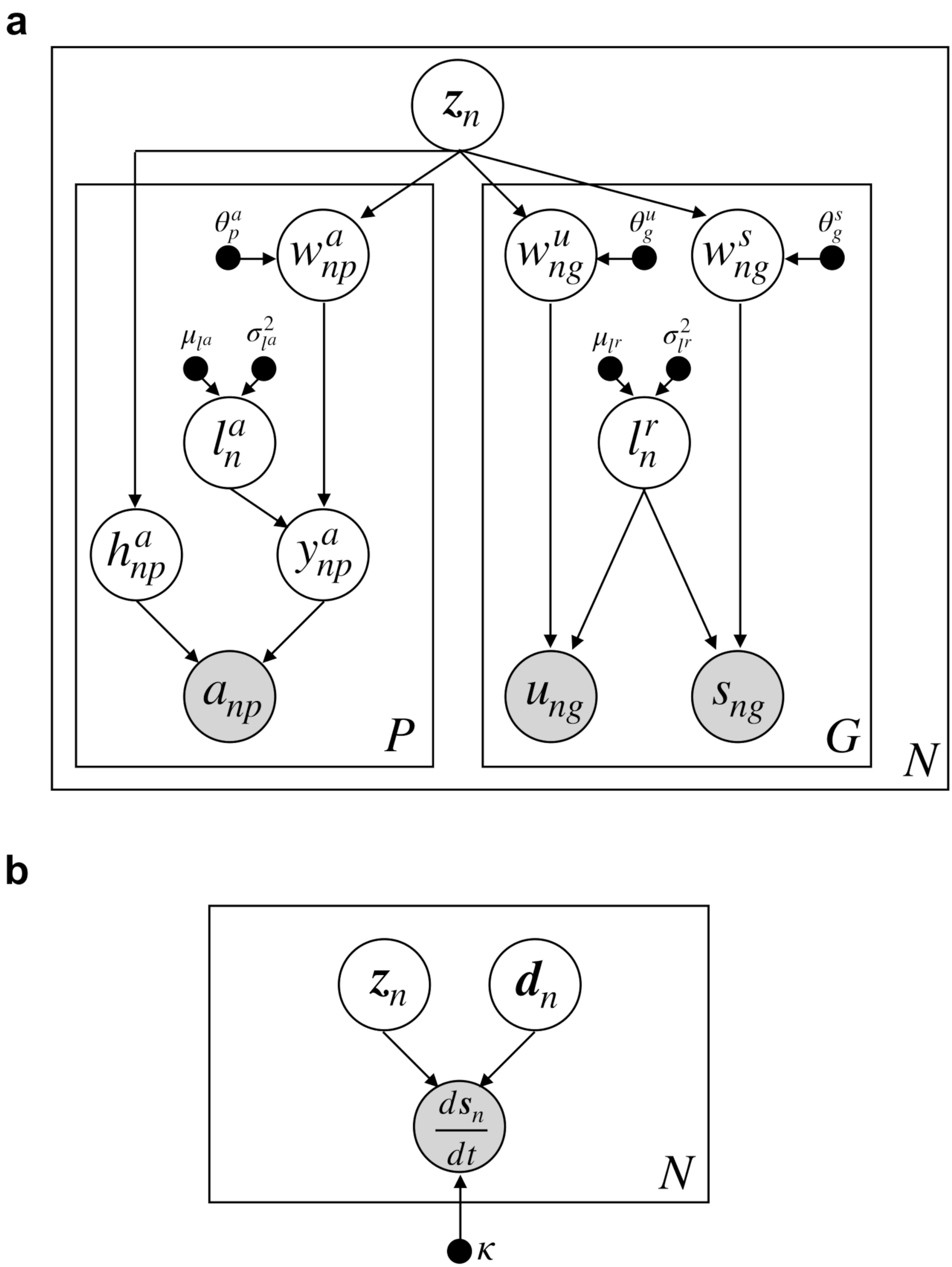

Figure S1 Graphical model of mmVelo. a, Graphical model of mmVelo for inferring cell state. **b**, Graphical model of mmVelo for inferring cell state dynamics. Detailed model descriptions and notations are in the Methods section.

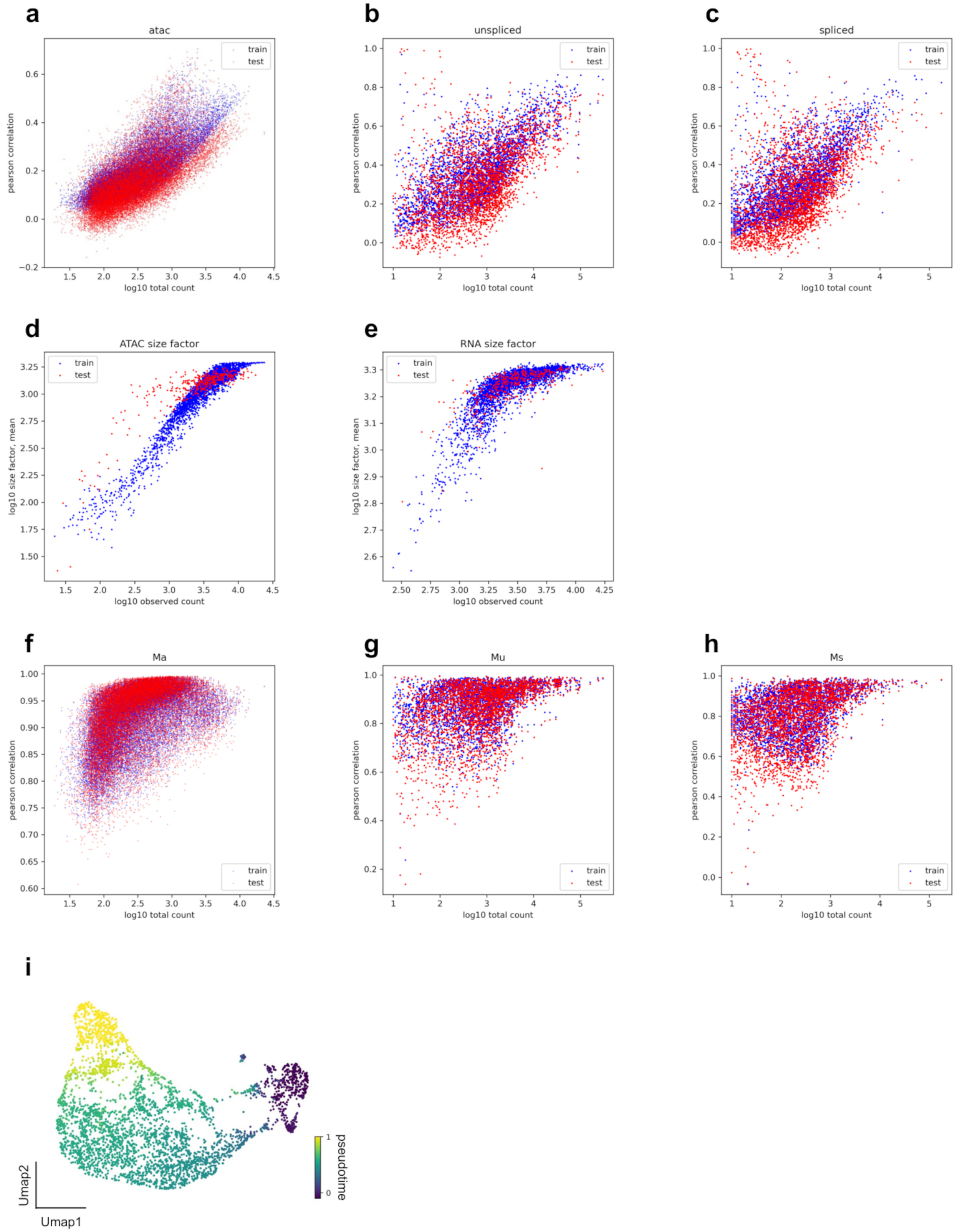

Figure S2 mmVelo model performance in embryonic mouse brain dataset, Related to Figure2. a-c, Scatter plot representing the peak-wise/gene-wise correlation between imputed counts in cell state inference and observed counts of ATAC (**a**), unspliced (**b**), and spliced (**c**). **d-e**, Scatter plot representing the cell-wise correlation between inferred size factor in cell state inference and observed library sizes of ATAC (**d**) and RNA (**e**). **f-h**, Scatter plot representing the peak-wise/gene-wise correlation between imputed smoothed profiles and observed moments of ATAC (**f**), unspliced (**g**), and spliced (**h**). **i**, UMAP plots colored by pseudotime. Pseudotime was estimated by diffusion pseudotime, using cell states inferred by mmVelo as inputs.

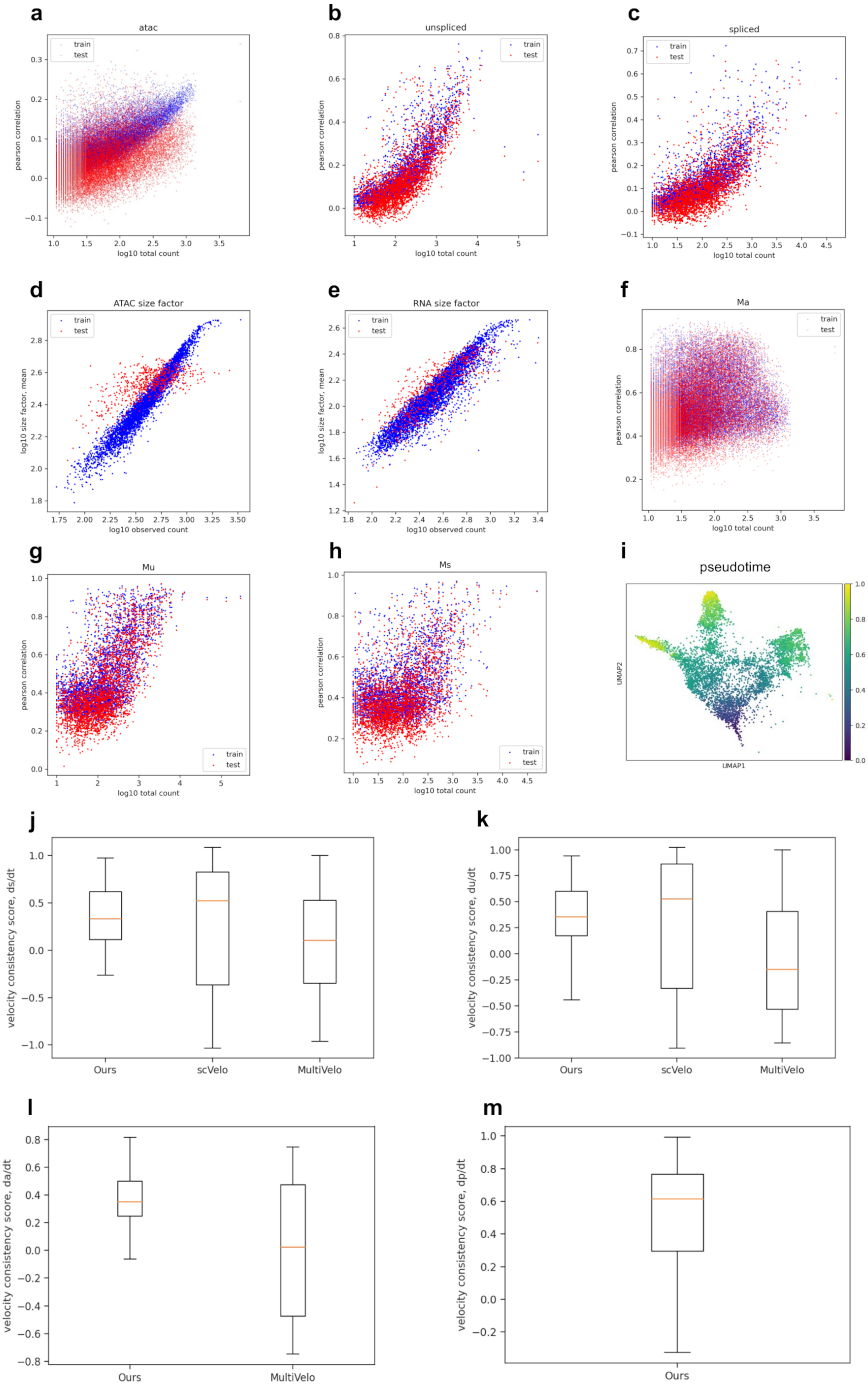

Figure S3 mmVelo model performance in mouse hair follicle development dataset, Related to Figure3. a-c, Scatter plot representing the peak-wise/gene-wise correlation between imputed counts in cell state inference and observed counts of ATAC (**a**), unspliced (**b**), and spliced (**c**). **d-e**, Scatter plot representing the cell-wise correlation between inferred size factor in cell state inference and observed library sizes of ATAC (**d**) and RNA (**e**). **f-h**, Scatter plot representing the peak-wise/gene-wise correlation between imputed smoothed profiles and observed moments of ATAC (**f**), unspliced (**g**), and spliced (**h**). **i**, UMAP plots colored by pseudotime. Pseudotime was estimated by diffusion pseudotime, using cell states inferred by mmVelo as inputs. **j-m**, Box plots of velocity consistency score by mmVelo(left), scVelo (middle) and MultiVelo (right) on spliced mRNA velocity (**j**), unspliced mRNA velocity (**k**), gene-wise aggregated chromatin velocity (**l**) and peak-wise chromatin velocity (**m**). In (**l**) scVelo, and in (**m**) scVelo and MultiVelo, are not included because these methods cannot infer the velocity in these modalities. Velocity consistency score was calculated in hair shaft-cuticle/cortex lineage cells. Detailed descriptions about benchmarking are in the Methods section.

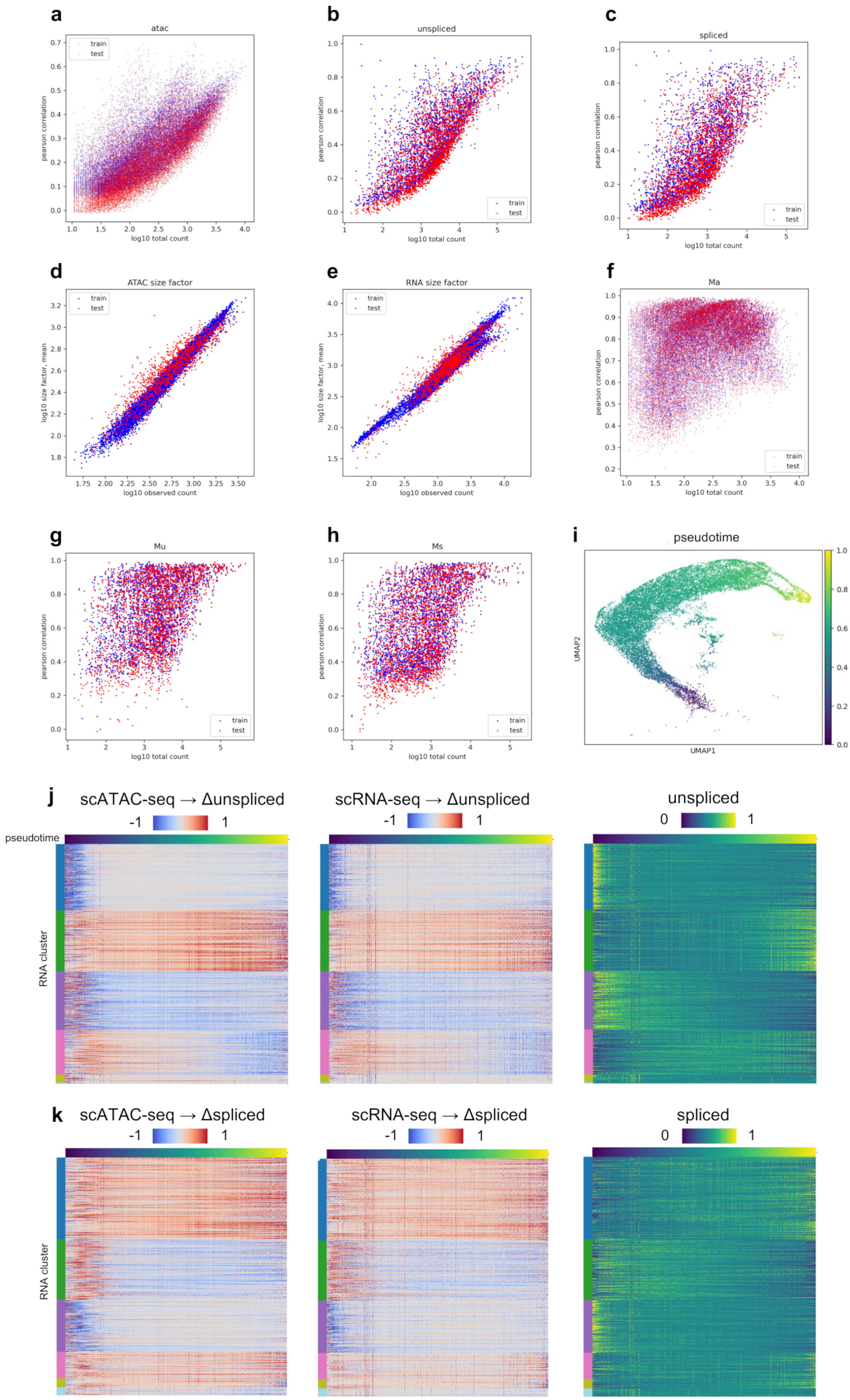

Figure S4 mmVelo model performance in human cortical development dataset, Related to Figure6. a-c, Scatter plot representing the peak-wise/gene-wise correlation between imputed counts in cell state inference and observed counts of ATAC (**a**), unspliced (**b**), and spliced (**c**). **d-e**, Scatter plot representing the cell-wise correlation between inferred size factor in cell state inference and observed library sizes of ATAC (**d**) and RNA (**e**). **f-h**, Scatter plot representing the peak-wise/gene-wise correlation between imputed smoothed profiles and observed moments of ATAC (**f**), unspliced (**g**), and spliced (**h**). **i**, UMAP plots of excitatory neuron lineage cells colored by pseudotime. Pseudotime was estimated by diffusion pseudotime, using cell states inferred by mmVelo as inputs. **j-k**, Heatmaps of unspliced mRNA velocity (**j**) and spliced mRNA velocity (**k**) inferred from scATAC-seq (left) and from scRNA-seq (middle), and observed mRNA (right) along pseudotime axis in GluN lineage.

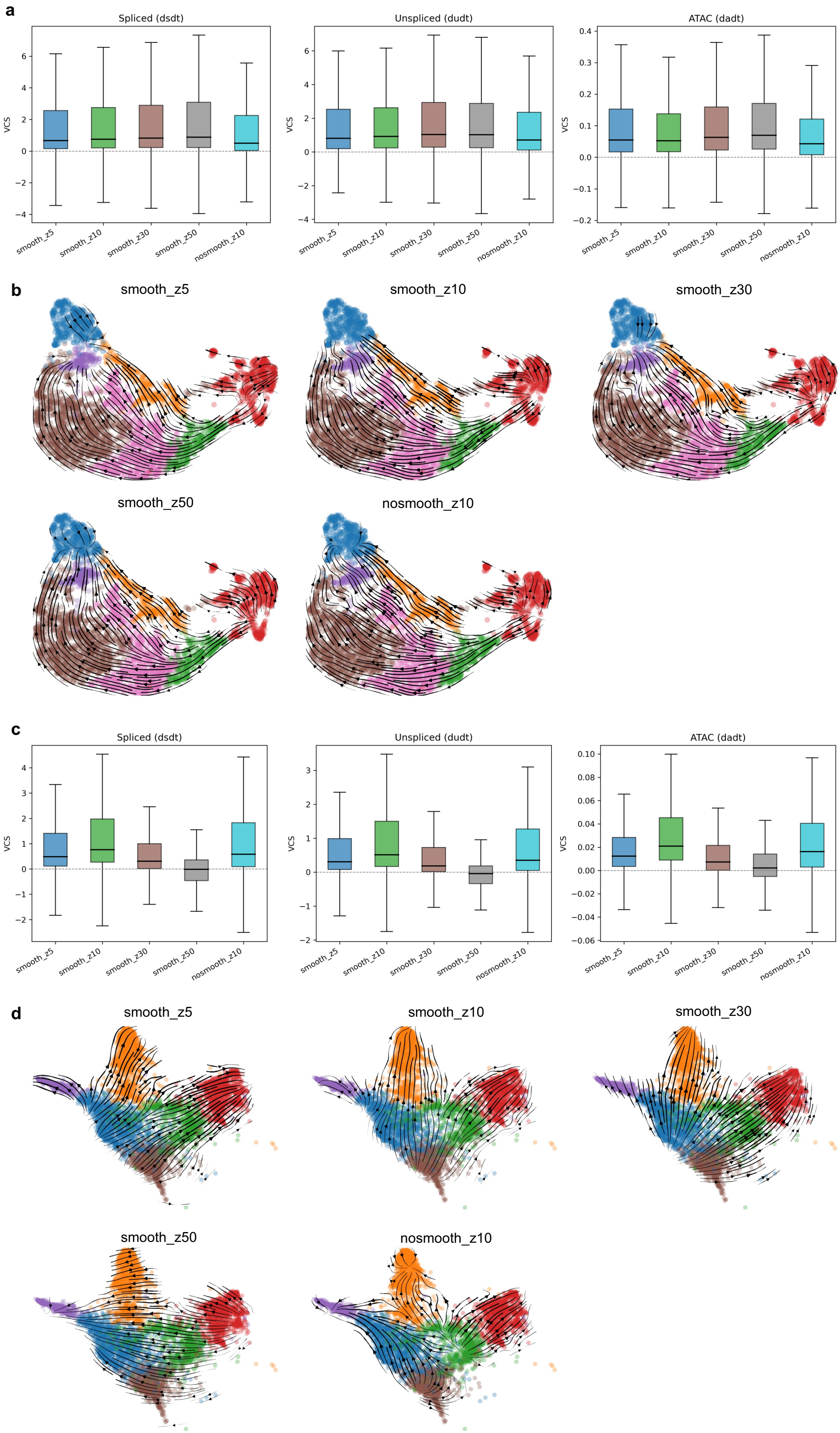

Figure S5 Sensitivity analysis of latent dimensionality and smoothing. a. Box plots of velocity consistency scores for spliced mRNA (left), unspliced mRNA (middle), and chromatin (right) velocities across different experimental settings in the embryonic mouse brain dataset. **b**. UMAP coordinates with stream plots across different experimental settings in the embryonic mouse brain dataset. Dots are colored by cell type. **c**. Box plots of velocity consistency scores for spliced mRNA (left), unspliced mRNA (middle), and chromatin (right) velocities across different experimental settings in the mouse hair follicle dataset. **d**. UMAP coordinates with stream plots across different experimental settings in the mouse hair follicle dataset. Dots are colored by cell type.

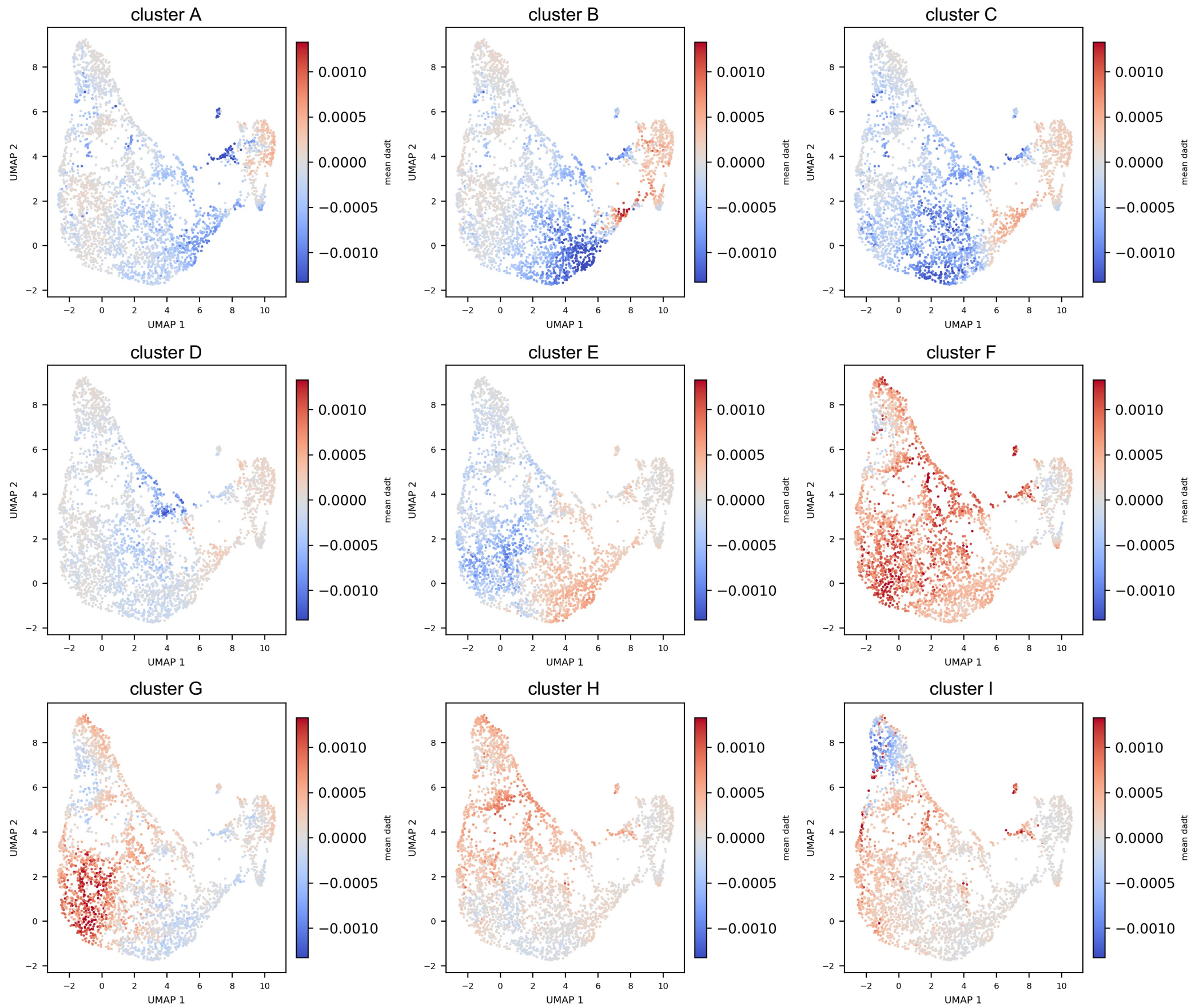

Figure S6 Chromatin velocity landscape across peak clusters, Related to Figure2. UMAP coordinates of cells colored by chromatin velocity averaged within each peak cluster. Each panel (**A–I**) corresponds to a distinct peak cluster.
